# The Efficacy of “Foundations,” a Digital Mental Health App to Improve Mental Well-being During COVID-19: Proof-of-Principle Randomized Controlled Trial

**DOI:** 10.2196/30976

**Published:** 2022-07-01

**Authors:** Silvina Catuara-Solarz, Bartlomiej Skorulski, Iñaki Estella-Aguerri, Claudia Bibiana Avella-Garcia, Sarah Shepherd, Emily Stott, Nicola R Hemmings, Aleix Ruiz de Villa, Laura Schulze, Sophie Dix

**Affiliations:** 1 Koa Health Barcelona Spain

**Keywords:** mental well-being, digital health, cognitive behavioral therapy, positive psychology, insomnia, COVID-19, mental health, mobile app, anxiety, health app

## Abstract

**Background:**

Against a long-term trend of increasing demand, the COVID-19 pandemic has led to a global rise in common mental disorders. Now more than ever, there is an urgent need for scalable, evidence-based interventions to support mental well-being.

**Objective:**

The aim of this proof-of-principle study was to evaluate the efficacy of a mobile-based app in adults with self-reported symptoms of anxiety and stress in a randomized control trial that took place during the first wave of the COVID-19 pandemic in the United Kingdom.

**Methods:**

Adults with mild to severe anxiety and moderate to high levels of perceived stress were randomized to either the intervention or control arm. Participants in the intervention arm were given access to the Foundations app for the duration of the 4-week study. All participants were required to self-report a range of validated measures of mental well-being (10-item Connor-Davidson Resilience scale [CD-RISC-10], 7-item Generalized Anxiety Disorder scale [GAD-7], Office of National Statistics Four Subjective Well-being Questions [ONS-4], World Health Organization-5 Well-Being Index [WHO-5]) and sleep (Minimal Insomnia Scale [MISS]) at baseline and at weeks 2 and 4. The self-reported measures of perceived stress (10-item Perceived Stress Score [PSS-10]) were obtained weekly.

**Results:**

A total of 136 participants completed the study and were included in the final analysis. The intervention group (n=62) showed significant improvements compared to the control group (n=74) on measures of anxiety, with a mean GAD-7 score change from baseline of –1.35 (SD 4.43) and –0.23 (SD 3.24), respectively (t_134_=1.71, P=.04); resilience, with a mean change in CD-RISC score of 1.79 (SD 4.08) and –0.31 (SD 3.16), respectively (t_134_=–3.37, P<.001); sleep, with a mean MISS score change of –1.16 (SD 2.67) and –0.26 (SD 2.29), respectively (t_134_=2.13, P=.01); and mental well-being, with a mean WHO-5 score change of 1.53 (SD 5.30) and –0.23 (SD 4.20), respectively (t_134_=–2.16, P=.02), within 2 weeks of using Foundations, with further improvements emerging at week 4. Perceived stress was also reduced within the intervention group, although the difference did not reach statistical significance relative to the control group, with a PSS score change from baseline to week 2 of –2.94 (SD 6.84) and –2.05 (SD 5.34), respectively (t_134_= 0.84, P=.20).

**Conclusions:**

This study provides a proof of principle that the digital mental health app Foundations can improve measures of mental well-being, anxiety, resilience, and sleep within 2 weeks of use, with greater effects after 4 weeks. Foundations therefore offers potential as a scalable, cost-effective, and accessible solution to enhance mental well-being, even during times of crisis such as the COVID-19 pandemic.

**Trial Registration:**

OSF Registries osf.io/f6djb; https://osf.io/vm3xq

## Introduction

### Background

Mental illness is a highly prevalent and complex public health issue. The total number of people with any mental health disorder reached 792 million in 2017 [1]. Moreover, according to the World Health Organization, the number of people with common mental disorders (CMD) such as mild to severe depression and anxiety is globally increasing over time [2]. These figures are worrying since mental health conditions account for a greater burden of disease based on years lived with disability [3] and they also are the largest cost driver in health care, estimated to reach over US $2.2 trillion and to rise to nearly US $6 trillion by 2030 [4].

Within the context of the COVID-19 pandemic, the prospects of the mental health status of society have become even more concerning. Numerous reports have highlighted that during the COVID-19 pandemic, poor mental health has been exacerbated globally [5,6]. In the United Kingdom, the population prevalence of clinically significant levels of mental distress rose from 18.9% (2018-2019) to 27.3% in April 2020 [7]. A recent study in the United States showed a 3-fold increase in depressive symptoms during the COVID-19 epidemic compared with the previous rate [8]. More recently, a meta-analysis of 66 studies with 221,970 participants reported an overall pooled prevalence of depression, anxiety, distress, and insomnia of 31.4%, 31.9%, 41.1%, and 37.9%, respectively [9]. Taken together, the magnitude of the socioeconomic burden of CMD illustrates that the current model of mental health care has yet to be fully optimized. Thus, better, more easily scalable interventions are urgently needed.

Digital technologies have shown great potential to offer scalable, easy-to-access, and timely solutions to increase the delivery of psychotherapeutic interventions and evidence-based recommendations for self-care and self-management [10,11]. During the COVID-19 pandemic, demand for these technologies has increased on a global scale and the use of mental health apps has risen exponentially. Although some apps have been shown to have positive effects on mental well-being [10,12,13], concerns remain regarding the level of credibility and the robustness of evidence underlying the majority of the thousands of available apps on the market [10,14]. More efforts toward the proper demonstration of their efficacy are needed if digital apps are to offer an adjunctive method to reduce the prevalence and impact of CMD across the world, and to support the growing mental health crisis surrounding the COVID-19 pandemic.

### Objectives

The aim of this 4-week proof-of-principle study was to test the efficacy of a digital intervention delivered via a mobile app, named Foundations, in comparison to a no-intervention control group on a range of psychological measures, including anxiety, mental well-being, resilience, sleep, and stress. More specifically, we aimed to assess the efficacy of Foundations in improving mental well-being. The study took place during the months of April and May of 2020 throughout the first outbreak of the COVID-19 pandemic in the United Kingdom.

Foundations includes a plethora of interventions and psychoeducational content that are scientifically robust (ie, cognitive behavioral therapy [CBT], meditation). Based on this, in combination with preliminary user research, we hypothesized that participants in the intervention group would show significant improvements compared with the control group in the following targeted areas of well-being after 4 weeks: (1) anxiety, (2) mental well-being, (3) resilience, (4) sleep, and (5) stress.

## Methods

### Ethics Approval

This study protocol involving human participants was General Data Protection Regulation–compliant and developed in accordance with Alpha Health’s ethics framework and principles [15], which are compliant with the World Medical Association’s Declaration of Helsinki [16]. All participants provided informed, electronic consent to share their data, including for publication of the results, prior to their enrollment in the study. Due to unforeseen logistical reasons, the study was not preregistered; however, all statistical testing was performed by a data analyst who remained blind to treatment assignment. Furthermore, the authors confirm that all ongoing studies for the Foundations app are registered.

### Trial Design

**Figure 1:**
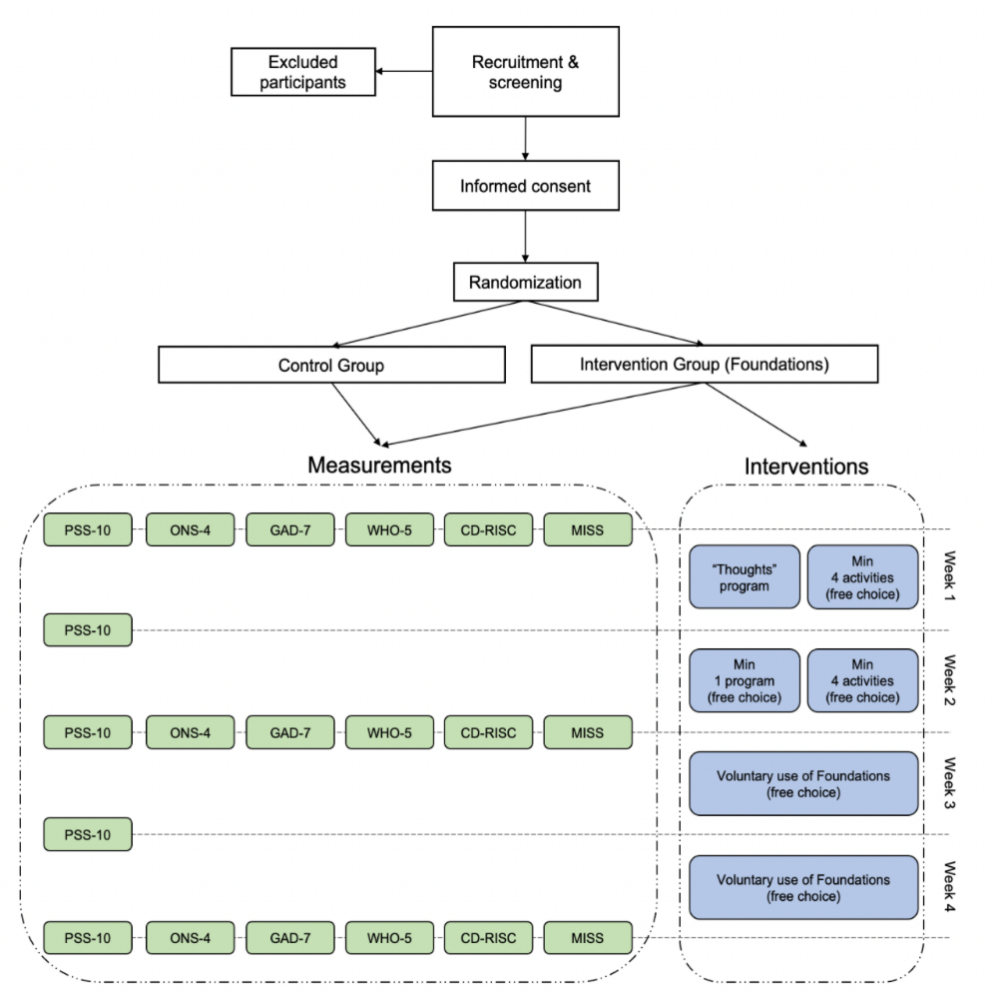
Overview of the study design. PSS-10: 10-item Perceived Stress Score; ONS-4: Office of National Statistics Four Subjective Well-being Questions; GAD-7: 7-item Generalized Anxiety Disorder scale; WHO-5: World Health Organization-5 Well-Being Index; CD-RISC: Connor-Davidson Resilience Scale; MISS: Minimal Insomnia Scale.

This proof-of-concept study was a 2-armed randomized controlled trial (RCT) comparing an app-based intervention (Foundations) to a nonintervention control group. At the time of the study, the app was named Evermind. It was rebranded in November 2020 and we refer to it as its current name (Foundations) throughout this paper. Anxiety, sleep, resilience, and mental well-being were assessed at baseline, and at weeks 2 and 4 of the study, and perceived stress was assessed weekly (at baseline through weeks 1 and 4; Figure 1). The study started on April 22, 2020, and ended on May 20, 2020. Of note, at the start of the study, the United Kingdom was at the first peak of the COVID-19 pandemic, and the reported numbers of positive cases and deaths were in decline at the end of the study [17].

### Participants

#### Recruitment

Study participants were recruited through a research company specializing in study recruitment between the first 2 weeks of April, according to a screening based on the following predefined criteria for inclusion and exclusion. Eligible participants were between 30 and 50 years old, owned a smartphone and regularly used apps, were fluent in English, and had been employed for at least 3 months in the United Kingdom. In addition, participants were considered eligible for the study if they showed moderate to high levels of perceived stress (10-item Perceived Stress Score [PSS-10]>13 [18,19]), mild to severe anxiety (7-item Generalized Anxiety Disorder scale [GAD-7] score of 5-18 [20]), and no to moderate sleep problems (Minimal Insomnia Scale [MISS] score of 0-8 [21]).

Candidates were excluded from the study in case of current pregnancy, current high-stress event (eg, family bereavement with the exception of a COVID-19 crisis), current psychotherapeutic treatment or counselling, current diagnosis of psychiatric illness (with the exception of depression) by a specialist/secondary care, regular use of mental health apps, or a recent (3 months) change in medication for mood disorders.

#### Assessment of Eligibility and Randomization

Eligible participants were randomly allocated (1:1) to either the intervention group or control group. Assignment of participants to the groups was performed with a computer-based algorithm (Python Software Foundation) that generated randomly permuted blocks, which were stratified by gender (male or female) and age (30-40 years or 40-50 years), and were balanced regarding the degree of perceived stress (“moderate perceived stress,” PSS-10 score of 13-26, or “high perceived stress,” PSS-10 score of 27-40) and sleep disturbances (“no sleep disturbances,” MISS score of 0-4, or “moderate sleep disturbances,” MISS score of 5-8). Furthermore, participants’ baseline scores on the GAD-7 were analyzed for statistical differences and were rerandomized if needed. The randomization analysis was performed by a statistician who remained blinded to the identity of the groups.

### Study Conditions

#### Intervention Group

**Figure 2:**
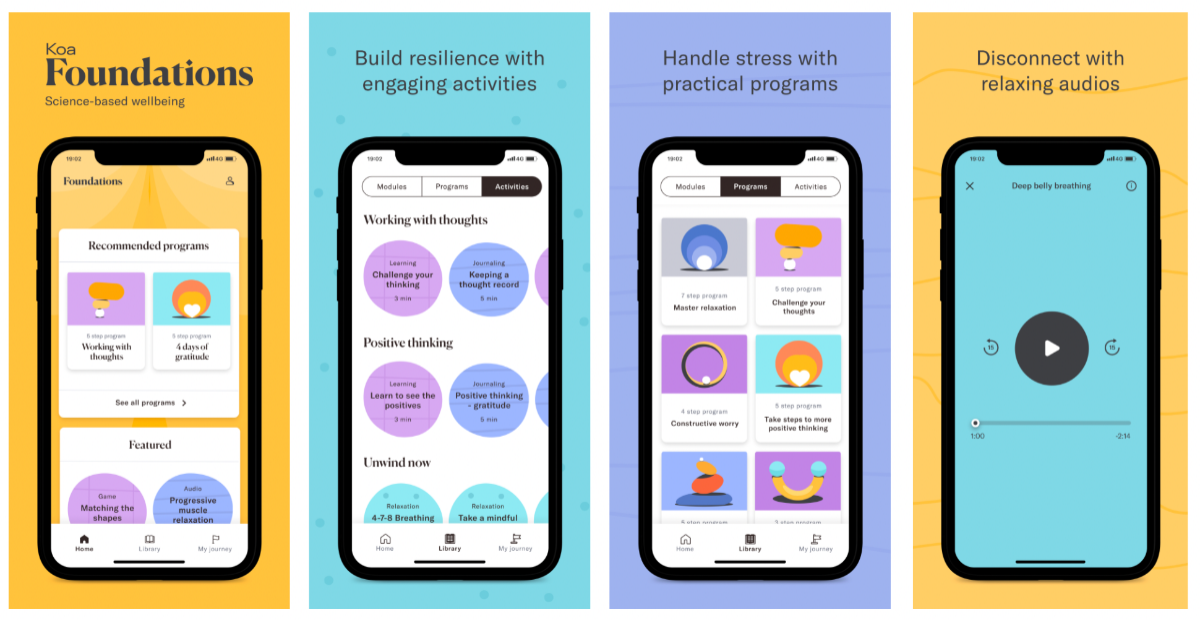
Screenshots of the Foundations app.

After randomization, the intervention group received access and instructions to download the Foundations app. The intervention app (Foundations) comprised interventions and psychoeducational content aimed at decreasing stress and promoting mental well-being (Figure 2). Content was organized into activities (ie, units of content) and could take a variety of formats, which are listed in Table 1. They all have a brief (typically 1-2 sentences) introduction and end with a closing sentence. Activities were either *in the moment* or part of a *program*. A *program* is a locked sequence of activities that is delivered in daily steps designed to teach a skill such as healthy sleep behaviors, positive psychology, working with thoughts, or relaxation techniques (see Table 2). *In the moment* activities were not part of a locked sequence of activities and could be accessed at any time. These activities included sleep meditations, articles, and mindfulness. Programs and activities were organized into a library of themed modules.

**Table 1:** Activity formats and descriptions of the content delivered in Foundations.

Activity	Number of activities	Type of content	Description
Slides	12	Psychoeducation	Comprises individual screens of 1-2 sentences that the user swipes through. Usually 10-20 screens
Article/blog	13	Psychoeducation and tips	User scrolls through to read. Typically 0.25-1 of A4 page in length
Add record	10	Journaling/reflection	User can add free text (eg, thought records or gratitude journaling)
Label record	5	Journaling/reflection	User selects a record created by the “add record” feature and can choose a theme label
Question record	5	Journaling/reflection	User selects a previous record and is asked a series of questions about the record. There is a free-text box for the user to write in
Record review	4	Journaling/reflection	Log of record entries
Audios	17	Mindfulness/meditations	Mindful meditations (5-8 min), sleep meditations (30 min), relaxation techniques
Ambient sounds	8	Ambient sounds	30-min relaxation sounds and soundscapes (eg, waves, rain)
Quiz	6	Interactive psychoeducation	Reinforces psychoeducation with two-choice answers
Game	1	Spatial working memory game	The user has to recall spatial sequences

**Table 2:** Description of programs and the number of activities within each program.

Program name	Number of activities	Description
Become a breathing master	3	Teaches the skill of diaphragmatic breathing
Relax your body and mind	2	Teaches the skill of progressive muscle relaxation
Working with thoughts	5	CBT^a^-based journaling and reflection. Includes psychoeducation on cognitive distortions and questions to balance unhelpful thoughts
Positive thinking	5	Gratitude journaling on achievements, reasons to be thankful, and people
Healthy sleep habits	9	CBT psychoeducation on healthy sleep habits and sleep hygiene
Break your bad sleep habits	4	CBT interactive psychoeducation on breaking bad habits
Take control of your sleep	9	CBT sleep scheduling
Constructive worry for sleep	4	Introduces constructive worry to put worries aside before bed
3 days to improve your self-esteem	10	CBT-based journaling and psychoeducation on automatic thoughts and balancing thoughts about oneself
Boost your confidence	3	Identify strengths

^a^CBT: cognitive behavioral therapy.

The study lasted 4 weeks and all participants started the study at the same time. During the first 2 weeks of the study, the participants were required to perform a minimum number of activities and programs. In week 1, participants were instructed to complete the “Working with thoughts” program, consisting of journaling and CBT interventions focused on ameliorating cognitive distortions and unhelpful ruminative patterns, and a minimum of 4 free-choice activities. During week 2, participants had to complete 1 program of their choosing and a minimum of 4 other activities (free choice). During weeks 3 and 4, participants were free to use the app as little or as much as they wished with complete free choice of activities and programs.

For the first 2 weeks of the research period, participants were encouraged to use Foundations via daily text messages. The messages were written and sent by the research manager via the messaging service WhatsApp and included wording such as:

Good morning! If you were able to start the programme yesterday then please do another activity of your choice from the Library page. If you weren’t able to start the programme yesterday, please do the first day of the Working with thoughts programme.

Remember, if you need to contact us at any point, the best way to get in touch is via email at: hello@evermind.health.

#### Control Group

The control group completed the same questionnaires at the same time points as the intervention group. At the end of the study, all participants were provided optional access to the Foundations app (waitlist control condition).

#### Incentives

At the end of the study, each participant received a monetary incentive as compensation for their involvement in the trial. As compensation was tied to the participants’ time, participants in the control group received £35 (US $50) and those in the intervention group received £85 (US $115).

### Outcome Measures

#### Overview

All participants were invited to fill out questionnaires via an online platform (Google Form) to assess their mental well-being on a weekly basis starting with the first day of the study (baseline). All participants, regardless of group, were sent the link to the online platform via the messaging service WhatsApp at the same time. Perceived stress was assessed weekly, whereas all other measures were assessed every 2 weeks (ie, baseline, 2 weeks, and 4 weeks). All outcome measures were treated as continuous variables. Each measure and the associated questionnaire are detailed below.

#### Anxiety

Anxiety levels were assessed through the GAD-7 scale [20], a 7-item questionnaire that measures the severity of the subject’s anxiety over the previous week.

#### Sleep Problems

The MISS was used to examine sleep problems in the sample and their evolution across the study [21]. The MISS includes 3 items that cover issues of initiating sleep, waking up in the night, and not feeling refreshed in the morning.

#### Resilience

Resilience levels were assessed by the Connor-Davidson Resilience Scale (CD-RISC-10), which includes 10 items that assess the individual’s ability to cope successfully with adversity [22].

#### Mental Well-being

To assess current mental well-being, the World Health Organization-5 Well-Being Index (WHO-5) questionnaire was administered to the study participants. Each of the 5 items is scored with a Likert scale ranging from 0 to 5 (at no time, some of the time, less than half of the time, more than half of the time, most of the time, all of the time, respectively) [23].

In addition, the United Kingdom Office of National Statistics questions on well-being (ONS-4) scale was used to measure subjective well-being [24]. Each of the questions in this scale is aimed at measuring a different aspect of well-being: life satisfaction, worthwhileness, happiness, and anxiety, which are each rated by the subject from 0 (not at all) to 10 (completely). These questions are not designed to provide an aggregate score, but rather to illustrate different aspects of perceived well-being.

#### Perceived Stress

The degree to which participants perceived their life situations as stressful was assessed using the PSS-10 [18,19]. The time frame selected for questions was the past week, which enabled examination of weekly effects of the intervention. It should be noted that participants were able to access the PSS-10 within the app whenever they liked. These additional data were not evaluated as part of the study.

### Statistical Analyses

#### Power

A power analysis based on published data and previous pilot studies [25] was performed (using the “pwr” R package) to estimate the required sample size for the study. The estimated sample size was at least 78 participants in each arm providing 0.8 power to detect an effect size of Cohen *d*=0.4 with an α of .05.

#### Data Analysis

Two principal sets of analyses were performed on each of the outcome measures. The first set of analyses, which we denote by *within-group analyses*, sought to determine whether there was a significant change within each group compared to the measure at the start of the study (baseline). Within-group paired two-tailed *t* tests were used for pre-post intervention assessments for each group. Statistical significance was set at P<.05. Bonferroni correction for multiple comparisons was performed adjusting the significance level to 1.25% for PSS-10 (significance level of 5% divided by 4 measures in time, baseline to weeks 1-4) and 2.5% (significance level of 5% divided by 2 measures in time, baseline to week 4) for the rest of the outcome measures.

The second set of analyses, denoted as *between-group analyses,* sought to determine whether the change from baseline (Δ) was equivalent in both groups. The analysis was performed using linear mixed models (LMMs) incorporating group (intervention or control), time, and group×time interaction terms, including a change score of 0 at the baseline time point and modeling participant as a random effect. Significance of the group variable was assessed using the likelihood ratio test. A confirmatory set of analyses was performed using an independent two-tailed *t* test on the differences of each group’s scores in each measure at a given time point from their baseline scores (Δ). Statistical significance was set at P<.05.

## Results

### Participants

**Figure 3:**
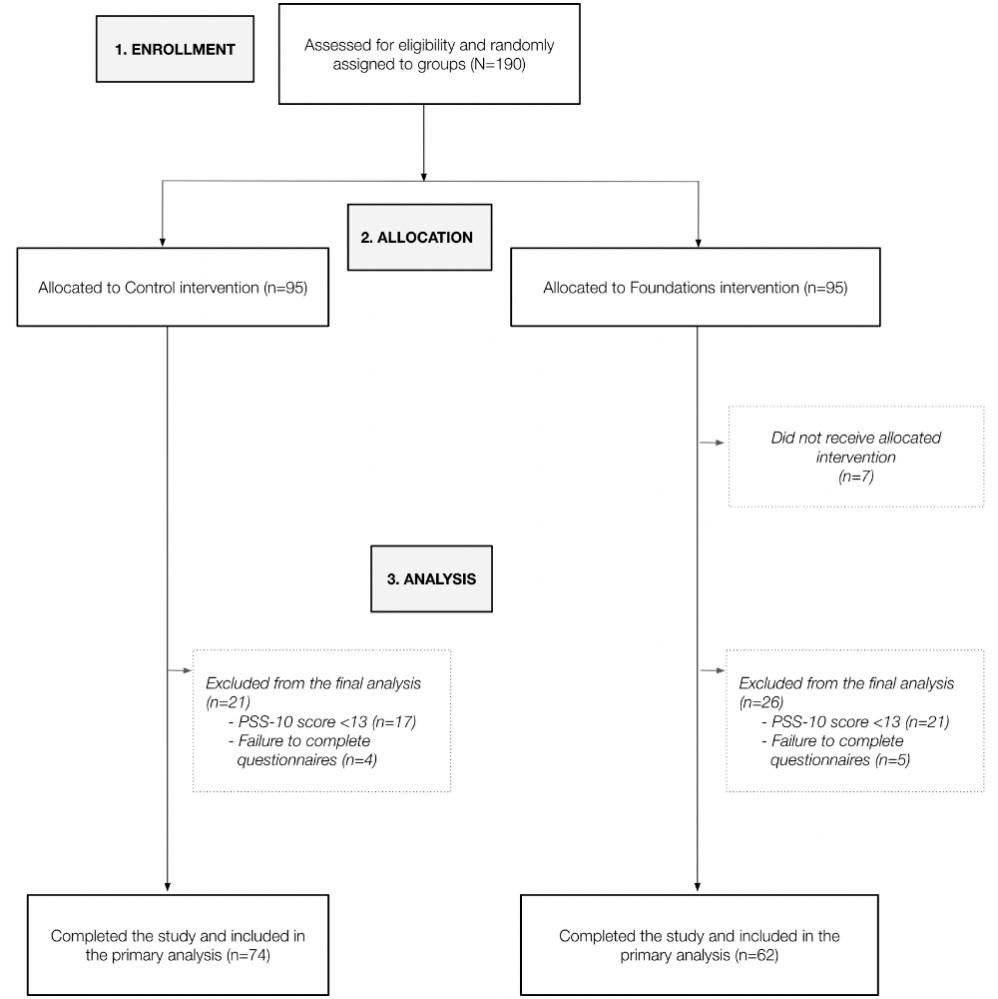
CONSORT (Consolidated Standards of Reporting Trials) flowchart of participants. PSS-10: 10-item Perceived Stress Score.

From April 2020 to May 2020, 190 participants were enrolled in the study and randomized to either the intervention group (n=95) or control group (n=95). Of the 95 participants in the intervention group, 7 failed to complete the study (ie, did not use the app as required) and were excluded from the primary analysis. A further 9 participants (5 in the intervention group and 4 in the control group) were excluded due to missing data (failed to complete the outcome measure questionnaires). An additional 38 participants (21 in the intervention group and 17 in the control group) were excluded from the analysis due to a calculation error of the PSS-10 at screening (PSS-10 score<13). Due to the study’s single-blind design, this error was not identified until after the study was completed. Of the remaining participants, 74 (79%) from the control group and 62 (65%) from the intervention group completed the study and were analyzed for the primary outcome and secondary outcome measures (Figure 3).

Table 3 provides the baseline demographics of the study participants (N=136). Participants in the intervention (n=62) and control (n=74) groups did not differ significantly with respect to gender, age, nor their levels of mental well-being at baseline.

The values for the within-group and between-group analyses at different points in time for each of the outcome measures are shown in Table 4 and Table 5, respectively. In the following, we discuss the results for each outcome metric as well as for the subgroup analysis and engagement. As described in the Methods section, for within-group analyses, significance thresholds were adjusted to account for multiple comparisons (Bonferroni correction) using a significance threshold of α=.025 for all scales excluding the PSS-10, which had a significance threshold of α=.0125.

**Table 3:** Characteristics of study participants at baseline.

Characteristic		Foundation group (n=62)	Control group (n=74)	Effect size (*d*)	P value	Clinical interpretation
**Gender**						
	Females, n	33	40	N/A^a^	.32	N/A
	Males, n	29	34	N/A	.48	N/A
Age (years), mean (SD)		40.58 (6.08)	39.49 (6.13)	N/A	.30	N/A
PSS-10^b^, mean (SD)		19.19 (4.12)	19.05 (3.58)	–0.04 (n^c^)	0.58	moderate stress
GAD-7^d^, mean (SD)		8.06 (3.83)	6.91 (3.28)	–0.32 (small)	0.97	mild anxiety
WHO-5^e^, mean (SD)		11.35 (4.29)	12 (4.23)	0.15 (n)	0.81	poor well-being
ONS-4^f^, mean (SD)		22.63 (5.14)	23.64 (5.82)	0.18 (n)	0.85	N/A
CD-RISC^g^, mean (SD)		23.27 (5.76)	24.91 (4.96)	0.31 (small)	0.96	problems in coping with stress or bouncing back from adversity
MISS^h^, mean (SD)		4.82 (2.3)	4.6 (2.51)	–0.09 (n)	0.7	no sleep problems

^a^N/A: not applicable.

^b^PSS-10: 10-item Perceived Stress Score.

^c^n: negligible or no effect.

^d^GAD-7: 7-item Generalized Anxiety Disorder scale.

^e^WHO-5: World Health Organization-5 Well-Being Index.

^f^ONS-4: Office of National Statistics Four Subjective Well-being Questions.

^g^CD-RISC: Connor-Davidson Resilience Scale.

^h^MISS: Minimal Insomnia Scale.

**Table 4:** Within-group analyses showing the change from baseline on the outcome measures at each time point (week 2 and week 4).

Outcome measure			Intervention group									Control group						
			Mean (SD)		*t (df*=61)		P value^a^		Effect size (*d*)		Mean (SD)			*t* (*df*=73)		P value^a^		Effect size (*d*)
**GAD-7^b^**																		
	Baseline	8.06 (3.83)		—^c^		—		—		6.92 (3.28)			—		—		—	
	Week 2	6.71 (4.79)		–2.41		<.001		–0.31		6.69 (3.92)			–0.61		.27		–0.06	
	Week 4	6.02 (4.29)		–3.69		<.001		–0.50		6.14 (3.88)			–1.92		.03		–0.22	
**MISS^d^**																		
	Baseline	4.82 (2.30)		—		—		—		4.61 (2.51)			—		—		—	
	Week 2	3.66 (2.28)		–3.43		<.001		–0.51		4.35 (2.61)			–0.96		.17		–0.10	
	Week 4	3.15 (1.90)		–5.31		<.001		–0.79		4.05 (1.72)			–2.03		.02		–0.21	
**CD-RISC-10^e^**																		
	Baseline	23.27 (5.76)		—		—		—		24.91 (4.97)			—		—		—	
	Week 2	25.06 (5.47)		3.45		<.001		0.31		24.59 (5.02)			–0.84		.80		–0.06	
	Week 4	25.66 (5.48)		4.07		<.001		0.42		25.19 (5.78)			0.58		.28		0.05	
**WHO-5^f^**																		
	Baseline	11.35 (4.29)		—		—		—		12.00 (4.23)			—		—		—	
	Week 2	12.89 (5.10)		2.28		.01		0.32		11.77 (4.38)			–0.47		.68		–0.05	
	Week 4	13.95 (4.45)		–3.85		<.001		0.59		12.12 (4.64)			0.25		.40		0.03	
**ONS-4^g^ Life satisfaction**																		
	Baseline	5.69 (1.53)		—		—		—		6.08 (1.76)			—		—		—	
	Week 2	6.66 (1.64)		3.79		<.001		0.61		6.69 (3.92)			1.33		.09		0.15	
	Week 4	6.95 (1.32)		5.41		<.001		0.88		6.61 (1.57)			2.60		.006		0.32	
**ONS-4 Worth**																		
	Baseline	6.34 (1.94)		—		—		—		6.30 (1.70)			—		—		—	
	Week 2	6.82 (1.89)		1.69		.05		0.25		6.42 (1.78)			0.64		.26		0.07	
	Week 4	7.06 (1.56)		2.64		.005		0.41		6.59 (1.70)			1.48		.07		0.17	
**ONS-4 Happiness**																		
	Baseline	5.95 (1.51)		—		—		—		6.16 (1.72)			—		—		—	
	Week 2	6.55 (1.87)		2.32		.01		0.35		6.35 (1.53)			0.99		.16		0.12	
	Week 4	7.08 (1.55)		4.99		<.001		0.74		6.47 (1.78)			1.39		.08		0.18	
**ONS-4 Anxiety**																		
	Baseline	4.65 (1.66		—		—		—		5.09 (2.00)			—		—		—	
	Week 2	5.13 (2.50)		1.64		.05		0.22		5.42 (2.25)			1.38		.09		0.15	
	Week 4	5.08 (2.77)		1.24		.11		0.19		5.12 (2.42)			0.09		.46		0.01	
**PSS-10^h^**																		
	Baseline	19.19 (4.12)		—		—		—		19.05 (3.58)			—		—		—	
	Week 1	18.32 (5.27)		–1.35		.09		–0.18		17.59 (5.03)			–2.95		.002		–0.32	
	Week 2	16.26 (6.20)		–3.38		<.001		–0.55		17.00 (5.50)			–3.31		<.001		–0.43	
	Week 3	15.65 (5.55)		–4.79		<.001		–0.72		17.01 (5.93			–3.04		.002		–0.40	
	Week 4	15.53 (5.82)		–4.86		<.001		–0.72		16.38 (5.72)			–3.99		<.001		–0.55	

^a^Comparisons were made for each time point relative to the baseline level; significance thresholds have been adjusted to account for multiple comparisons (Bonferroni): P<.025 for all outcomes except for PSS-10, which was set to P<.012.

^b^GAD-7: 7-item Generalized Anxiety Disorder scale.

^c^not applicable.

^d^MISS: Minimal Insomnia Scale.

^e^CD-RISC-10: Connor-Davidson Resilience Scale.

^f^WHO-5: World Health Organization-5 Well-Being Index.

^g^ONS-4: Office of National Statistics Four Subjective Well-being Questions.

^h^PSS-10: 10-item Perceived Stress Score.

**Table 5:** Between-group analyses showing the change of outcome measures (Δ) from baseline at each time point (week 2 and week 4).

Outcome measure			Intervention Δ, mean (SD)		Control Δ, mean (SD)		Mean difference	*t* (*df*=134)		P value		Effect size (*d*)
**GAD-7^a^**												
	Week 2	–1.35 (4.43)		–0.23 (3.24)		1.13		1.71	.04		0.29	
	Week 4	–2.05 (4.37)		–0.78 (3.52)		1.26		1.87	.03		0.32	
**MISS^b^**												
	Week 2	–1.16 (2.67)		–0.26 (2.29)		0.90		2.13	.02		0.37	
	Week 4	–1.68 (2.49)		–0.55 (2.35)		1.12		2.70	.004		0.47	
**CD-RISC^c^**												
	Week 2	1.79 (4.08)		–0.31 (3.16)		–2.10		–3.37	<.001		–0.58	
	Week 4	2.39 (4.62)		0.28 (4.24)		–2.10		–2.78	.003		–0.48	
**WHO-5^d^**												
	Week 2	1.53 (5.30)		–0.23 (4.20)		–1.76		–2.16	.02		–0.37	
	Week 4	2.59 (5.32)		0.12 (4.20)		–2.46		–3.05	.001		–0.52	
**ONS-4^e^ life satisfaction**												
	Week 2	0.97 (2.01)		0.24 (1.58)		–0.72		–2.36	.01		–0.41	
	Week 4	1.26 (1.53)		0.53 (1.75)		–0.73		–2.38	.009		–0.41	
**ONS-4 worth**												
	Week 2	0.48 (2.25)		0.12 (1.63)		–0.36		–1.09	.14		–0.19	
	Week 4	0.73 (2.17)		0.297 (1.76)		–0.43		–1.28	.10		–0.22	
**ONS-4 happiness**												
	Week 2	0.596 (2.02)		0.19 (1.64)		–0.41		–1.29	.10		–0.22	
	Week 4	1.13 (1.78)		0.31 (1.92)		–0.82		–2.56	.006		–0.44	
**ONS-4 anxiety**												
	Week 2	0.48 (2.32)		0.32 (2.02)		–0.16		–0.43	.33		–0.07	
	Week 4	0.44 (2.77)		0.03 (2.48)		–0.41		–0.91	.18		–0.16	
**PSS-10^f^**												
	Week 1	–0.87 (5.09)		–1.45 (4.25)		–0.58		–0.73	.77		–0.13	
	Week 2	–2.94 (6.84)		–2.05 (5.34)		0.88		0.84	.20		0.15	
	Week 3	–3.55 (5.84)		–2.04 (5.77)		1.51		1.51	.07		0.26	
	Week 4	–3.66 (5.93)		–2.68 (5.77)		0.99		0.98	.16		0.17	

^a^GAD-7: 7-item Generalized Anxiety Disorder scale.

^b^MISS: Minimal Insomnia Scale.

^c^CD-RISC: Connor-Davidson Resilience Scale.

^d^WHO-5: World Health Organization-5 Well-Being Index

^e^ONS-4: Office of National Statistics Four Subjective Well-being Questions.

^f^PSS-10: 10-item Perceived Stress Score.

### Anxiety (GAD-7)

Within-group *t* tests for those in the intervention arm (Foundations) showed that GAD-7 scores reduced significantly compared with those at baseline at both week 2 and week 4. In contrast, there was no change in GAD-7 scores in the control group when comparing baseline to week 2 (Table 4).

**Figure 4:**
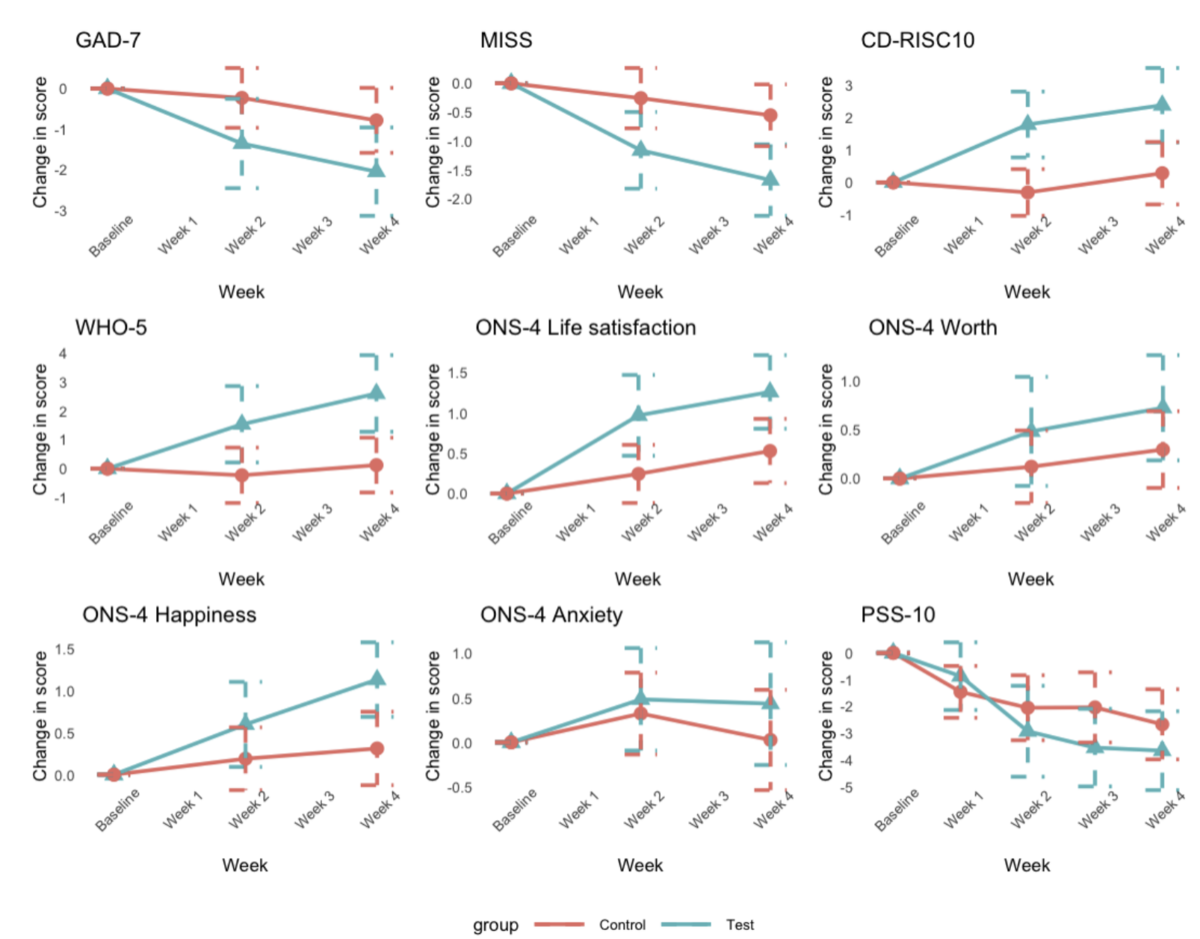
Between-group analyses showing the change from baseline on outcome measures at each time point. Data points represent the mean and bars are standard errors. PSS-10: 10-item Perceived Stress Score; ONS-4: Office of National Statistics Four Subjective Well-being Questions; GAD-7: 7-item Generalized Anxiety Disorder scale; WHO-5: World Health Organization-5 Well-Being Index; CD-RISC: Connor-Davidson Resilience Scale; MISS: Minimal Insomnia Scale.

For the between-group comparisons, LMM analysis of the change from baseline (Δ) showed a significant global effect of the intervention (P=.03), where the main impact seems to be at week 4 with P=.05 for the interaction term (see Tables S1 and S2 in Multimedia Appendix 1). Posthoc comparisons confirmed significant differences at weeks 2 and 4 (Figure 4, Table 5), such that the intervention group had lower GAD-7 scores than the control group. These data suggest that Foundations reduced anxiety within 4 weeks of use.

### Sleep (MISS)

Within-group analysis of the MISS score showed that the score in the intervention group significantly improved both at week 2 and week 4. In comparison, within-group analysis of the control group showed no significant change in the MISS score from baseline to week 2 or from baseline to week 4 (see Table 4).

Between-group LMM analyses of the change from baseline revealed a significantly greater improvement in MISS in the intervention group compared to the control group (P=.01). Even though there was a global decrease of MISS at week 4, interaction terms showed significant results within weeks 2 and 4 (see Tables S3 and S4 in Multimedia Appendix 1). Posthoc comparisons confirmed significant differences at weeks 2 and 4 (Figure 4, Table 5), such that the intervention group had lower MISS scores (higher sleep quality) than the control group (also see Tables S3 and S4 in Multimedia Appendix 1). These data suggest that Foundations improves sleep within 2 weeks of use.

### Resilience (CD-RISC-10)

Within-group analyses of the two groups showed significant improvement in resilience scores for the intervention group at both weeks 2 and 4, but no effect in the control group (Table 4).

There were significant between-group effects in the analysis of the change from baseline, such that the intervention group showed a greater change in score than the control group (P<.001). Moreover, we found a significant positive effect (interaction terms) within the intervention group at both weeks 2 and 4 (see Tables S5 and S6 in Multimedia Appendix 1). Posthoc comparisons confirmed significant differences at weeks 2 and 4 (Figure 4, Table 5), such that the intervention group had higher CD-RISC-10 scores (higher resiliency) compared with those of the control group. These data suggest that Foundations enhances resilience within 2 weeks of use.

### Mental Well-being

#### WHO-5 Scores

Within-group analyses of each group by time showed that the WHO-5 score significantly increased in the intervention group at week 4 but not at week 2. Among controls, there was no significant change at either time point (Table 4).

Group differences were revealed in the LMM between-group analysis (see Tables S7 and S8 of Multimedia Appendix 1) of the change from baseline: the intervention group showed a greater change from baseline than the control group (P=.03) in both weeks 2 and 4. Posthoc comparisons confirmed these significant differences at weeks 2 and 4 (Figure 4, Table 5), such that the intervention group had higher WHO-5 scores (higher quality of life) compared with those of the control group. These data suggest that Foundations can enhance well-being within 2 weeks of use, as measured by the WHO-5.

#### ONS-4 Questionnaire

The ONS-4 questionnaire comprises four independent questions on life satisfaction, worth, happiness, and anxiety (the scale for the latter has been reversed in the analysis, so that a larger score means a greater positive impact on the participants). The within-group analyses of the intervention group showed improvements in life satisfaction at week 2 and week 4, improvements in worth at week 4, and improvements in happiness at week 2 and week 4. No improvement in anxiety was found at either time point. In contrast, in the control group, only the measure of life satisfaction at week 4 showed a significant improvement compared to baseline (Table 4).

All LMM analyses revealed only overall significant effects on ONS satisfaction and happiness (P=.002 and P=.008, respectively; see Tables S9 and S13 in Multimedia Appendix 1), with inconclusive effects on ONS on worth and anxiety (see Tables S11 and S15 in Multimedia Appendix 1). On ONS satisfaction, we found a significant effect at both 2 and 4 weeks, whereas on ONS happiness, we only found significant effects at week 4 (see interaction terms in Tables S9 and S13 in Multimedia Appendix 1). These results were confirmed by a posthoc *t* test, which showed higher scores in the intervention group than in the control group on the measures of life satisfaction and happiness (Figure 4, Table 5). These data suggest that the intervention group experienced improvement in the sense of life satisfaction and happiness compared to the control group.

#### Perceived Stress (PSS-10)

In contrast to the other measures, perceived stress was measured weekly. Within-group analyses showed that the PSS-10 score was significantly lower at 2, 3, and 4 weeks, but not at week 1, compared to that measured at baseline for the intervention group (Table 4). However, the control group also showed significantly lower perceived stress levels at each of the four time points (Table 4).

In the same way, between-group analyses of the change from baseline showed no statistically significant result. The likelihood ratio test on LMM showed a P value of .19 (see Tables S17 and S18 in Multimedia Appendix 1), and no significant group differences were observed using *t* tests (Table 5). These data suggest that perceived stress was reduced across the course of the study, but this reduction was no greater in the intervention group than in the control group.

### Subclass Analyses

Additional analyses were performed with the factors of gender and age included on each of the outcome measures for both within-group and between-group comparisons. No statistically significant differences were observed, due to the fact that the size of groups was small. See Table S19 in Multimedia Appendix 1 for the age and gender interaction terms for each of the metrics.

### Engagement With the App

The participants in the intervention group presented an average usage of the app of 18 days out of the 28 (SD 5.1) total days of the study period (median days active: 17). On average, participants engaged with the app 15.25 minutes per day (median minutes per day 13.93, SD 9.66). During the study period, users tried an average of 29 distinct activities and 3.9 programs. However, no correlation was found between the total amount of engagement with the app and the difference from baseline to 4 weeks in any of the outcome measures (all P>.05).

Research suggests that the level of engagement with a digital intervention impacts the outcomes [26]. However, this was not the case in our study, which may be due to the mechanisms of action taking place outside of the app in which the user is encouraged by the app to complete a healthy behavior. Once a behavior is learned or a skill is developed, the user may not need to engage with the app to experience the benefits (eg, a user can maintain a healthy sleep routine without accessing the app every night).

## Discussion

### Principal Findings

The aim of the study was to investigate the efficacy of a mobile app, Foundations, in improving mental well-being in adults with moderate to high levels of stress and anxiety. Given the timing of the study (April 2020 to May 2020), our secondary aim was to assess the efficacy of Foundations in mitigating the mental health challenges surrounding the COVID-19 pandemic.

Encouragingly, results of this proof-of-principle study confirmed four out of five of our hypotheses by demonstrating that the use of Foundations can significantly improve measures of (1) anxiety, (2) resilience, (3) well-being, and (4) sleep relative to a control group within 2 weeks of use, with greater effects after 4 weeks. In contrast to our final hypothesis, perceived stress was reduced within the intervention group, although the results did not reach statistical significance relative to the control group.

### Comparison With Prior Work

The results of this study contribute to a limited number of RCTs examining the efficacy of mental health apps that are commercially available. Encouragingly, our findings are consistent with published meta-analyses that have affirmed the efficacy of digital interventions over control conditions in improving mental well-being. For example, a meta-analysis of 18 RCTs reported that smartphone interventions were significantly more efficacious in reducing depressive symptoms in comparison to both waitlist and active control groups [27]. Congruent with results from a recent meta-analysis of 66 RCTs of mobile apps for mental health, we found a greater reduction in anxiety among participants in the intervention versus control group as well as greater improvements in resilience and overall mental well-being [28].

However, our results are at odds with previous RCTs reporting greater reductions in stress in active versus control interventions [29,30]. These results were surprising given the positive results on measures of anxiety, resilience, and well-being. This lack of a statistically significant effect is due to an improvement overall in the control group across the period of the study. There are several potential factors that may have contributed to this finding. First, the study started at the peak of the COVID-19 pandemic in the United Kingdom (April 2020). The reported numbers of positive cases and deaths were reduced by approximately 40% during the 4 weeks of the study [17]. Further, as the population adjusted to lockdown measures, perceived stress levels may have naturally reduced. However, it is not clear why this would impact perceived stress to a greater extent to, for example, anxiety and resilience. Another factor may be the frequency of testing. The PSS-10 was assessed on a weekly basis as opposed to every 2 weeks in the case of all other measures. Foundations’ users were also able to take the PSS-10 whenever they liked within the app; these were voluntary assessments, and the data were not evaluated. It is therefore possible that survey fatigue or more frequent insight into stress levels impacted perceived stress.

With regard to intervention features, Foundations differentiates from many of the apps that have published efficacy data in that it offers a breadth of interactive content delivered. For example, Sleepio [31] provides an internet-based CBT course targeted at individuals with insomnia, and Woebot [32] provides CBT-directed mental health via an artificial intelligence–powered chatbot for individuals struggling with poor mental health symptoms. Foundations provides a wider array of support across the mental health spectrum via a number of methodologies (CBT, acceptance and commitment training, positive psychology, and sleep science). Although it is unclear whether single- versus multi-intervention apps differ in efficacy, at the very least, multi-intervention apps such as Foundations offer users flexibility and variety in the content and functionalities they can choose from, which has been shown to increase app engagement and likability [33].

Despite the rapid upscaling of digital mental health interventions during the COVID-19 pandemic, literature on the efficacy of these technologies is rather sparse at present. To the best of our knowledge, this study is one of the few RCTs investigating the efficacy of a mental health app during the COVID-19 pandemic [34,35]. The importance of this research is two-fold. First, the results from this study demonstrate that Foundations could add a scalable efficacious digital intervention to support mental well-being that can be accessed at any time. Second, these findings may help to inform the rationale and design of future studies and digital health technologies. Going forward, it will be critical to maintain this momentum as the mental health consequences of the pandemic could be severe and long-lasting.

### Limitations

Several important limitations of this study should be recognized. Perhaps the most important limitation is the notable number of participants excluded from the primary analysis due to a calculation error of the PSS-10 at screening. It remains uncertain whether a larger sample of patients would result in outcomes that differ from those observed; however, the sample size for the final analysis (N=136) is comparable to those reported in the literature on digital interventions for mental well-being [27,28].

Second, it is possible that the daily scheduled broadcast messages received by participants in the Foundations group contributed to a placebo effect or to results that do not reflect real-world usage of the app. Conversely, it is also possible that the frequency of the notifications *hindered* engagement in some cases, as the amount of contact for optimizing user engagement likely varies across participants. However, it is notable that the size of the effect was greater at 4 weeks than at 2 weeks for all measures. Participants received no messages during weeks 3 and 4 other than the links to fill out the questionnaires. It therefore seems unlikely that the daily messaging could account for the efficacy observed in the study. However, the impact of the frequency and content of notifications on well-being outcomes remains to be fully elucidated and further studies may be warranted.

Another limitation of the study is the use of a monetary incentive for participation. Previous studies have shown that monetary incentives can increase engagement with wellness apps yet have no impact on the outcome of the study [12]. However, the true influence of the monetary incentive in this case is unknown.

Finally, it is necessary to acknowledge the limitations of a single-blind design, as used here. Only the data analysts were blind to the group assignment. The control group participants were aware they were taking part in an intervention study, but were only given access to the app after the study (and were not informed they would have access until study completion). The design of the study and the passive nature of the control group do not allow ruling out digital placebo effects in the intervention group derived from their expectations about the interventions [36]. Both groups, however, received the same messages and invitations to fill out the outcome questionnaires. It remains possible that insights into mental well-being through completion of the questionnaires, along with knowledge that they were participating in an intervention trial may have impacted outcome measures. Future studies may explore the use of alternative designs such as an active control or psychoeducation control group.

### Future Research

Before we can optimize the efficacy of digital health technologies such as Foundations, we must build a richer understanding of which interventions are most effective for which individual needs. It was not feasible with the sample size of the current study to evaluate the efficacy of individual components of the app with respect to symptom type and severity, and the content that the participant engaged with during the course of the study. An important question for future research is whether personalization of these interventions makes care more effective.

Similarly, it is unclear whether there was an effect of the level of engagement (ie, a dose effect). Although a satisfactory level of engagement was observed in this study, establishing a dose-response relationship between usage and improvement in mental well-being could prove useful for intervention personalization and bears further investigation in future.

A specific domain that has gained attention in recent years is that of work-related mental health. This is due to an increase of awareness of the magnitude and costs of this issue. Concretely, it has been reported that an outstanding 72% of employees of large organizations in the United Kingdom have disclosed an increase of CMD during 2019 [37]. Ill mental health in the workplace is associated with decreased productivity, early retirement, increased sickness absence, presenteeism (not working at capacity while at work), and staff turnover. All this translates into an estimated cost of over US $45 billion per year for companies, which has increased by 16% in the last few years [37]. Although this study examined the efficacy of Foundations in a working population, a truly rigorous investigation of its effects on workplace mental health would require a future study employing randomized, controlled allocation of participants to an intervention arm (Foundations) or placebo arm, both in the absence and presence of employment. At the very least, these preliminary observations provide evidence that working adults using the Foundations app can experience significant improvement in their mental well-being during a 2-week period.

Future research is also required to evaluate the long-term effects of Foundations on mental well-being both in terms of postintervention follow-up and longer sustained use of the app. Looking forward and if replicated in further studies, these results may have important implications for addressing the treatment gap in mental health care through the use of evidence-based digital interventions.

Overall, results from this study may help to propel the use of mobile apps such as Foundations to assume a more widespread role in both the promotion and maintenance of mental health. This could offer new possibilities to further optimize the efficacy of these technologies while removing obstacles for evidence-based mental health care.

### Conclusions

This proof-of-principle study demonstrates that Foundations can improve measures of anxiety, sleep, resilience, and mental well-being within 2 weeks of use, with a greater effect after 4 weeks. Foundations may therefore offer potential as a scalable, cost-effective intervention to enhance mental well-being even during a period of crisis such as the COVID-19 pandemic.

## Appendix

Multimedia Appendix 1 Supplementary Tables S1-S19.

Multimedia Appendix 2 CONSORT-eHEALTH checklist (V 1.6.2).

## Abbreviations

CBT: cognitive behavioral therapy
CD-RISC: Connor-Davidson Resilience Scale
CMD: common mental disorder
GAD-7: 7-item Generalized Anxiety Disorder scale
LMM: linear mixed model
MISS: Minimal Insomnia Scale
ONS-4: Office of National Statistics Four Subjective Well-being Questions
PSS-10: 10-item Perceived Stress Score
RCT: randomized controlled trial
WHO-5: World Health Organization-5 Well-Being Index

## References

[1] Dattani S, Ritchie H, Roser M (2021). Mental health. Our World in Data.

[2] (2017). Depression and other common mental disorders: global health estimates. World Health Organization.

[3] Wittchen H, Jacobi F, Rehm J, Gustavsson A, Svensson M, Jönsson B, Olesen J, Allgulander C, Alonso J, Faravelli C, Fratiglioni L, Jennum P, Lieb R, Maercker A, van Os J, Preisig M, Salvador-Carulla L, Simon R, Steinhausen H (2011). The size and burden of mental disorders and other disorders of the brain in Europe 2010. Eur Neuropsychopharmacol.

[4] Gustavsson A, Svensson M, Jacobi F, Allgulander C, Alonso J, Beghi E, Dodel R, Ekman M, Faravelli C, Fratiglioni L, Gannon B, Jones DH, Jennum P, Jordanova A, Jönsson L, Karampampa K, Knapp M, Kobelt G, Kurth T, Lieb R, Linde M, Ljungcrantz C, Maercker A, Melin B, Moscarelli M, Musayev A, Norwood F, Preisig M, Pugliatti M, Rehm J, Salvador-Carulla L, Schlehofer B, Simon R, Steinhausen H, Stovner LJ, Vallat J, Van den Bergh P, Van den Bergh P, van Os J, Vos P, Xu W, Wittchen HU, Jönsson B, Olesen J, CDBE 2010 Study Group (2011). Cost of disorders of the brain in Europe 2010. Eur Neuropsychopharmacol.

[5] Czeisler MÉ, Lane RI, Petrosky E, Wiley JF, Christensen A, Njai R, Weaver MD, Robbins R, Facer-Childs ER, Barger LK, Czeisler CA, Howard ME, Rajaratnam SM (2020). Mental health, substance use, and suicidal ideation during the COVID-19 pandemic - United States, June 24-30, 2020. MMWR Morb Mortal Wkly Rep.

[6] Vindegaard N, Benros ME (2020). COVID-19 pandemic and mental health consequences: systematic review of the current evidence. Brain Behav Immun.

[7] Pierce M, Hope H, Ford T, Hatch S, Hotopf M, John A, Kontopantelis E, Webb R, Wessely S, McManus S, Abel KM (2020). Mental health before and during the COVID-19 pandemic: a longitudinal probability sample survey of the UK population. Lancet Psychiatry.

[8] Ettman CK, Abdalla SM, Cohen GH, Sampson L, Vivier PM, Galea S (2020). Prevalence of depression symptoms in US adults before and during the COVID-19 pandemic. JAMA Netw Open.

[9] Wu T, Jia X, Shi H, Niu J, Yin X, Xie J, Wang X (2021). Prevalence of mental health problems during the COVID-19 pandemic: a systematic review and meta-analysis. J Affect Disord.

[10] Donker T, Petrie K, Proudfoot J, Clarke J, Birch M, Christensen H (2013). Smartphones for smarter delivery of mental health programs: a systematic review. J Med Internet Res.

[11] Naslund JA, Aschbrenner KA, Araya R, Marsch LA, Unützer J, Patel V, Bartels SJ (2017). Digital technology for treating and preventing mental disorders in low-income and middle-income countries: a narrative review of the literature. Lancet Psychiatry.

[12] Kawadler JM, Hemmings NR, Ponzo S, Morelli D, Bird G, Plans D (2020). Effectiveness of a smartphone app (BioBase) for reducing anxiety and increasing mental well-being: pilot feasibility and acceptability study. JMIR Form Res.

[13] Ponzo S, Morelli D, Kawadler JM, Hemmings NR, Bird G, Plans D (2020). Efficacy of the digital therapeutic mobile app BioBase to reduce stress and improve mental well-being among university students: randomized controlled trial. JMIR Mhealth Uhealth.

[14] Larsen ME, Huckvale K, Nicholas J, Torous J, Birrell L, Li E, Reda B (2019). Using science to sell apps: Evaluation of mental health app store quality claims. NPJ Digit Med.

[15] Ethical principles. Koa Health.

[16] World Medical Association (2013). World Medical Association Declaration of Helsinki: ethical principles for medical research involving human subjects. JAMA.

[17] (2020). United Kingdom Coronavirus. Worldometer.

[18] Cohen S, Kamarck T, Mermelstein R (1983). A global measure of perceived stress. J Health Soc Behav.

[19] Lee E (2012). Review of the psychometric evidence of the perceived stress scale. Asian Nurs Res (Korean Soc Nurs Sci).

[20] Spitzer RL, Kroenke K, Williams JBW, Löwe B (2006). A brief measure for assessing generalized anxiety disorder: the GAD-7. Arch Intern Med.

[21] Broman J, Smedje H, Mallon L, Hetta J (2008). The Minimal Insomnia Symptom Scale (MISS): a brief measure of sleeping difficulties. Ups J Med Sci.

[22] Campbell-Sills L, Stein MB (2007). Psychometric analysis and refinement of the Connor-davidson Resilience Scale (CD-RISC): validation of a 10-item measure of resilience. J Trauma Stress.

[23] Topp CW, Østergaard SD, Søndergaard S, Bech P (2015). The WHO-5 Well-Being Index: a systematic review of the literature. Psychother Psychosom.

[24] Dolan P, Metcalfe R (2012). Measuring subjective wellbeing: recommendations on measures for use by national governments. J Soc Pol.

[25] Baik SH, Fox RS, Mills SD, Roesch SC, Sadler GR, Klonoff EA, Malcarne VL (2019). Reliability and validity of the Perceived Stress Scale-10 in Hispanic Americans with English or Spanish language preference. J Health Psychol.

[26] Perski O, Blandford A, West R, Michie S (2017). Conceptualising engagement with digital behaviour change interventions: a systematic review using principles from critical interpretive synthesis. Transl Behav Med.

[27] Firth J, Torous J, Nicholas J, Carney R, Pratap A, Rosenbaum S, Sarris J (2017). The efficacy of smartphone-based mental health interventions for depressive symptoms: a meta-analysis of randomized controlled trials. World Psychiatry.

[28] Linardon J, Cuijpers P, Carlbring P, Messer M, Fuller-Tyszkiewicz M (2019). The efficacy of app-supported smartphone interventions for mental health problems: a meta-analysis of randomized controlled trials. World Psychiatry.

[29] Coelhoso CC, Tobo PR, Lacerda SS, Lima AH, Barrichello CRC, Amaro E, Kozasa EH (2019). A new mental health mobile app for well-being and stress reduction in working women: randomized controlled trial. J Med Internet Res.

[30] Fuller-Tyszkiewicz M, Richardson B, Little K, Teague S, Hartley-Clark L, Capic T, Khor S, Cummins RA, Olsson CA, Hutchinson D (2020). Efficacy of a smartphone app intervention for reducing caregiver stress: randomized controlled trial. JMIR Ment Health.

[31] Elison S, Ward J, Williams C, Espie C, Davies G, Dugdale S, Ragan K, Chisnall L, Lidbetter N, Smith K (2017). Feasibility of a UK community-based, eTherapy mental health service in Greater Manchester: repeated-measures and between-groups study of 'Living Life to the Full Interactive', 'Sleepio' and 'Breaking Free Online' at 'Self Help Services'. BMJ Open.

[32] Fitzpatrick KK, Darcy A, Vierhile M (2017). Delivering cognitive behavior therapy to young adults with symptoms of depression and anxiety using a fully automated conversational agent (Woebot): a randomized controlled trial. JMIR Ment Health.

[33] Alqahtani F, Orji R (2020). Insights from user reviews to improve mental health apps. Health Informatics J.

[34] Boucher EM, McNaughton EC, Harake N, Stafford JL, Parks AC (2021). The impact of a digital intervention (Happify) on loneliness during COVID-19: qualitative focus group. JMIR Ment Health.

[35] Fiol-DeRoque MA, Serrano-Ripoll MJ, Jiménez R, Zamanillo-Campos R, Yáñez-Juan AM, Bennasar-Veny M, Leiva A, Gervilla E, García-Buades ME, García-Toro M, Alonso-Coello P, Pastor-Moreno G, Ruiz-Pérez I, Sitges C, García-Campayo J, Llobera-Cánaves J, Ricci-Cabello I (2021). A mobile phone-based intervention to reduce mental health problems in health care workers during the COVID-19 pandemic (PsyCovidApp): randomized controlled trial. JMIR Mhealth Uhealth.

[36] Torous J, Firth J (2016). The digital placebo effect: mobile mental health meets clinical psychiatry. Lancet Psychiatry.

[37] Hampson E, Jacob A (2020). Mental health and employers: refreshing the case for investment. Deloitte.

