# Supplementary material for: The Efficacy of “Foundations,” a Digital Mental Health App to Improve Mental Well-being During COVID-19: Proof-of-Principle Randomized Controlled Trial
Source: JMIR Mhealth Uhealth. 2022 Jul 1;10(7):e30976. doi: 10.2196/30976 (PMC9255362; doi:10.2196/30976)
Supplement: Multimedia Appendix 1 [file mhealth_v10i7e30976_app1.docx]

Table S1. GAD model coefficients.

|  | Estimate | df | t value | p.value |
| --- | --- | --- | --- | --- |
| Intercept | 0.00 (0.37) | 333 | 0.000 | 1.000 |
| Group | 0.00 (0.55) | 333 | 0.000 | 1.0000 |
| Day 14 | -0.23 (0.43) | 268 | -0.536 | 0.5925 |
| Day 28 | -0.784 (0.43) | 268 | -1.828 | 0.0686 |
| Group:Day 14 | -1.125 (0.63) | 268 | -1.772 | 0.0776 |
| Group:Day 28 | -1.265 (0.63) | 268 | -1.992 | 0.0474 |

Table S2. GAD: likelihood ratio test.

| model | #parameters | log-likelihood | deviance | statistic | df | p.value |
| --- | --- | --- | --- | --- | --- | --- |
| Without group | 5 | -1032 | 2063 | NA | NA | NA |
| With group | 8 | -1027 | 2054 | 8.7 | 3 | 0.0336 |

Table S3. MISS - model coefficient

|  | Estimate | df | t value | p.value |
| --- | --- | --- | --- | --- |
| Intercept | 0 (0.23) | 313 | 0.000 | 1 |
| Group | 0 (0.34) | 313 | 0.000 | 1 |
| Day 14 | -0.257 (0.26) | 268 | -0.991 | 0.3224 |
| Day 28 | -0.554 (0.26) | 268 | -2.139 | 0.0333 |
| Group:Day 14 | -0.905 (0.38) | 268 | -2.358 | 0.0191 |
| Group:Day 28 | -1.123 (0.38) | 268 | -2.929 | 0.0037 |

Table S4. MISS: likelihood ratio test

| model | #parameters | log-likelihood | deviance | statistic | df | p.value |
| --- | --- | --- | --- | --- | --- | --- |
| Without group | 5 | -839.6 | 1679 | NA | NA | NA |
| With group | 8 | -831.5 | 1663 | 16.19 | 3 | 0.001 |

Table S5. CD-RISC model coefficients.

|  | Estimate | df | t value | p.value |
| --- | --- | --- | --- | --- |
| Intercept | 0 (0.38) | 335 | 0.000 | 1.000 |
| Group | 0 (0.57) | 335 | 0.000 | 1.000 |
| Day 14 | -0.311 (0.45) | 268 | -0.693 | 0.4886 |
| Day 28 | 0.284 (0.45) | 268 | 0.633 | 0.5272 |
| Group:Day 14 | 2.101 (0.66) | 268 | 3.165 | 0.0017 |
| Group:Day 28 | 2.103 (0.66) | 268 | 3.168 | 0.0017 |

Table S6. CD-RISC: likelihood ratio test

| model | #parameters | log-likelihood | deviance | statistic | df | p.value |
| --- | --- | --- | --- | --- | --- | --- |
| Without group | 5 | -1056 | 2112 | NA | NA | NA |
| With group | 8 | -1044 | 2088 | 24.2 | 3 | 0.000 |

Table S7*.* WHO-5 model coefficients.

|  | Estimate | df | t value | p.value |
| --- | --- | --- | --- | --- |
| Intercept | 0 (0.45) | 327 | 0.000 | 1.000 |
| Group | 0 (0.66) | 327 | 0.000 | 1.000 |
| Day 14 | -0.23 (0.52) | 268 | -0.446 | 0.6562 |
| Day 28 | 0.122 (0.52) | 268 | 0.236 | 0.8137 |
| Group:Day 14 | 1.762 (0.76) | 268 | 2.308 | 0.0218 |
| Group:Day 28 | 2.475 (0.76) | 268 | 3.242 | 0.0013 |

Table S8. WHO-5: likelihood ratio test.

| model | #parameters | log-likelihood | deviance | statistic | df | p.value |
| --- | --- | --- | --- | --- | --- | --- |
| Without group | 5 | -1115 | 2230 | NA | NA | NA |
| With group | 8 | -1106 | 2211 | 19.04 | 3 | 0.0003 |

Table S9.  ONS (satisfaction) model coefficients.

|  | Estimate | df | t value | p.value |
| --- | --- | --- | --- | --- |
| Intercept | 0 (0.17) | 325 | 0.000 | 1.000 |
| Group | 0 (0.25) | 325 | 0.000 | 1.000 |
| Day 14 | 0.243 (0.19) | 268 | 1.255 | 0.2106 |
| Day 28 | 0.527 (0.19) | 268 | 2.719 | 0.007 |
| Group:Day 14 | 0.724 (0.29) | 268 | 2.524 | 0.0122 |
| Group:Day 28 | 0.731 (0.29) | 268 | 2.546 | 0.0114 |

Table S10. ONS (satisfaction) likelihood ratio test.

| model | #parameters | log-likelihood | deviance | statistic | df | p.value |
| --- | --- | --- | --- | --- | --- | --- |
| Without group | 5 | -715.2 | 1430 | NA | NA | NA |
| With group | 8 | -707.7 | 1415 | 15.13 | 3 | 0.0017 |

Table S11.  ONS (worth) model coefficients

|  | Estimate | df | t value | p.value |
| --- | --- | --- | --- | --- |
| Intercept | 0 (0.18) | 324 | 0.000 | 1.000 |
| Group | 0 (0.27) | 324 | 0.000 | 1.000 |
| Day 14 | 0.122 (0.21) | 268 | 0.578 | 0.5635 |
| Day 28 | 0.297 (0.21) | 268 | 1.414 | 0.1586 |
| Group:Day 14 | 0.362 (0.31) | 268 | 1.163 | 0.2458 |
| Group:Day 28 | 0.429 (0.31) | 268 | 1.376 | 0.17 |

Table S12. ONS (worth) likelihood ratio test

| model | #parameters | log-likelihood | deviance | statistic | df | p.value |
| --- | --- | --- | --- | --- | --- | --- |
| Without group | 5 | -742.9 | 1486 | NA | NA | NA |
| With group | 8 | -740.9 | 1482 | 3.89 | 3 | 0.273 |

Table S13.  ONS (happiness) model coefficients.

|  | Estimate | df | t value | p.value |
| --- | --- | --- | --- | --- |
| Intercept | 0 (0.17) | 345 | 0.000 | 1.000 |
| Group | 0 (0.26) | 345 | 0.000 | 1.000 |
| Day 14 | 0.189 (0.21) | 268 | 0.908 | 0.3649 |
| Day 28 | 0.311 (0.21) | 268 | 1.491 | 0.1371 |
| Group:Day 14 | 0.408 (0.31) | 268 | 1.32 | 0.1879 |
| Group:Day 28 | 0.818 (0.31) | 268 | 2.65 | 0.0085 |

Table S14. ONS (happiness) likelihood ratio test.

| model | #parameters | log-likelihood | deviance | statistic | df | p.value |
| --- | --- | --- | --- | --- | --- | --- |
| Without group | 5 | -732.6 | 1465 | NA | NA | NA |
| With group | 8 | -726.7 | 1453 | 11.78 | 3 | 0.0082 |

Table S15. ONS (anxiety) model coefficients.

|  | Estimate | df | t value | p.value |
| --- | --- | --- | --- | --- |
| Intercept | 0 (0.23) | 361 | 0.000 | 1.000 |
| Group | 0 (0.34) | 361 | 0.000 | 1.000 |
| Day 14 | 0.324 (0.28) | 268 | 1.153 | 0.2499 |
| Day 28 | 0.027 (0.28) | 268 | 0.096 | 0.9235 |
| Group:Day 14 | 0.16 (0.42) | 268 | 0.383 | 0.702 |
| Group:Day 28 | 0.408 (0.42) | 268 | 0.981 | 0.3277 |

Table S16. ONS (anxiety) likelihood ratio test.

| model | #parameters | log-likelihood | deviance | statistic | df | p.value |
| --- | --- | --- | --- | --- | --- | --- |
| Without group | 5 | -840.7 | 1681 | NA | NA | NA |
| With group | 8 | -839.8 | 1680 | 1.64 | 3 | 0.6507 |

Table S17*.* PSS model coefficients.

|  | Estimate | df | t value | p.value |
| --- | --- | --- | --- | --- |
| Intercept | 0 (0.58) | 385 | 0.000 | 1.000 |
| Group | 0 (0.87) | 385 | 0.000 | 1.000 |
| Day 7 | -1.459 (0.62) | 536 | -2.341 | 0.0196 |
| Day 14 | -2.054 (0.62) | 536 | -3.295 | 0.0011 |
| Day 21 | -2.041 (0.62) | 536 | -3.273 | 0.0011 |
| Day 28 | -2.676 (0.62) | 536 | -4.292 | 0.000 |
| Group:Day 7 | 0.588 (0.92) | 536 | 0.637 | 0.5242 |
| Group:Day 14 | -0.881 (0.92) | 536 | -0.955 | 0.3402 |
| Group:Day 21 | -1.508 (0.92) | 536 | -1.633 | 0.1031 |
| Group:Day 28 | -0.986 (0.92) | 536 | -1.067 | 0.2863 |

Table S18. PSS - likelihood ratio test

| model | #parameters | log-likelihood | deviance | statistic | df | p.value |
| --- | --- | --- | --- | --- | --- | --- |
| With group | 7 | -1976 | 3953 | NA | NA | NA |
| With group | 12 | -1973 | 3945 | 7.43 | 5 | 0.1904 |

Table S19. Subgroup analysis.

| metric | p-value age | p-value gender |
| --- | --- | --- |
| cdrisk | 0.7629 | 0.4943 |
| gad | 0.09809 | 0.7381 |
| miss | 0.5837 | 0.9069 |
| ons | 0.6646 | 0.6461 |
| ons1 | 0.4746 | 0.7779 |
| ons2 | 0.8201 | 0.5014 |
| ons3 | 0.1524 | 0.4493 |
| ons4 | 0.4517 | 0.1384 |
| pss | 0.4751 | 0.4001 |
| who | 0.4782 | 0.5698 |
