# Supplementary material for: The Efficacy of “Foundations,” a Digital Mental Health App to Improve Mental Well-being During COVID-19: Proof-of-Principle Randomized Controlled Trial
Source: JMIR Mhealth Uhealth. 2022 Jul 1;10(7):e30976. doi: 10.2196/30976 (PMC9255362; doi:10.2196/30976)
Supplement: Multimedia Appendix 2 [file mhealth_v10i7e30976_app2.pdf]

CONSORT-EHEALTH Checklist V1.6.2 Report	Manuscript Number	30976
**(based on CONSORT-EHEALTH V1.6), available at [http://tinyurl.com/consort-ehealth-v1-6].**		
**Date completed**		
2/16/2022 11:39:04		
**by**		
Laura Schulze		
The Efficacy of “Foundations,” a Digital Mental Health App to Improve Mental Well-being During COVID-19: Proof-of-Principle Randomized Controlled Trial		
TITLE		
**1a-i) Identify the mode of delivery in the title**		
""Foundations," a Digital Mental Health App"		
**1a-ii) Non-web-based components or important co-interventions in title**		
Not applicable		
**1a-iii) Primary condition or target group in the title**		
Within the title: "a Digital Mental Health App to Improve Mental Well-being During COVID-19"<br><br>Included in the body of the article: "Adults with mild to severe anxiety and moderate to high levels of perceived stress"		
ABSTRACT		
**1b-i) Key features/functionalities/components of the intervention and comparator in the METHODS section of the ABSTRACT**		
"Adults with mild to severe anxiety and moderate to high levels of perceived stress were randomized to either the intervention or control arm. Participants in the intervention arm were given access to the Foundations app for the duration of the 4-week study."		
**1b-ii) Level of human involvement in the METHODS section of the ABSTRACT**		
**1b-iii) Open vs. closed, web-based (self-assessment) vs. face-to-face assessments in the METHODS section of the ABSTRACT**		
"All participants were required to self-report a range of validated measures of mental well-being (10-item Connor-Davidson Resilience scale [CD-RISC-10], 7-item Generalized Anxiety Disorder scale [GAD-7], Office of National Statistics Four Subjective Well-being Questions [ONS-4], World Health Organization-5 Well-Being Index [WHO-5]) and sleep (Minimal Insomnia Scale [MISS]) at baseline and at weeks 2 and 4. The self-reported measures of perceived stress (10-item Perceived Stress Score [PSS-10]) were obtained weekly."		
**1b-iv) RESULTS section in abstract must contain use data**		
"A total of 136 participants completed the study and were included in the final analysis. The intervention group (n=62) showed significant improvements compared to the control group (n=74)"		
**1b-v) CONCLUSIONS/DISCUSSION in abstract for negative trials**		
INTRODUCTION		
**2a-i) Problem and the type of system/solution**		
"The aim of this 4-week proof-of-principle study was to test the efficacy of a digital intervention delivered via a mobile app, named Foundations, in comparison to a no-intervention control group on a range of psychological measures, including anxiety, mental well-being, resilience, sleep, and stress. More specifically, we aimed to assess the efficacy of Foundations in improving mental well-being. The study took place during the months of April and May of 2020 throughout the first outbreak of the COVID-19 pandemic in the United Kingdom."		
**2a-ii) Scientific background, rationale: What is known about the (type of) system**		
"Foundations includes a plethora of interventions and psychoeducational content that are scientifically robust (ie, cognitive behavioral therapy [CBT], meditation). Based on this, in combination with preliminary user research, we hypothesized that participants in the intervention group would show significant improvements compared with the control group in the following targeted areas of well-being after 4 weeks: (1) anxiety, (2) mental well-being, (3) resilience, (4) sleep, and (5) stress."		
**Does your paper address CONSORT subitem 2b?**		
Please see response to Question 2a-i		
METHODS		
**3a) CONSORT: Description of trial design (such as parallel, factorial) including allocation ratio**		
"This proof-of-concept study was a 2-armed randomized controlled trial (RCT) comparing an app-based intervention (Foundations) to a nonintervention control group."<br><br>"Eligible participants were randomly allocated (1:1) to either the intervention group or control group."		
**3b) CONSORT: Important changes to methods after trial commencement (such as eligibility criteria), with reasons**		
Not applicable		
**3b-i) Bug fixes, Downtimes, Content Changes**		
Not applicable		
**4a) CONSORT: Eligibility criteria for participants**		
"Eligible participants were between 30 and 50 years old, owned a smartphone and regularly used apps, were fluent in English, and had been employed for at least 3 months in the United Kingdom. In addition, participants were considered eligible for the study if they showed moderate to high levels of perceived stress (10-item Perceived Stress Score [PSS-10]>13 [18,19]), mild to severe anxiety (7-item Generalized Anxiety Disorder scale [GAD-7] score of 5-18 [20]), and no to moderate sleep problems (Minimal Insomnia Scale [MISS] score of 0-8 [21]).<br><br>Candidates were excluded from the study in case of current pregnancy, current high-stress event (eg, family bereavement with the exception of a COVID-19 crisis), current psychotherapeutic treatment or counselling, current diagnosis of psychiatric illness (with the exception of depression) by a specialist/secondary care, regular use of mental health apps, or a recent (3 months) change in medication for mood disorders."		
**4a-i) Computer / Internet literacy**		
"Eligible participants ... owned a smartphone and regularly used apps"		
**4a-ii) Open vs. closed, web-based vs. face-to-face assessments:**		
Study participants were recruited online through a research company. After randomization, the intervention group received access and instructions via email to download the Foundations app.<br><br>"For the first 2 weeks of the research period, participants were encouraged to use Foundations via daily text messages."<br><br>All participants (intervention or control group) were required to complete (online) self-report questionnaires.		
**4a-iii) Information giving during recruitment**		
**4b) CONSORT: Settings and locations where the data were collected**		
"The study started at the peak of the COVID-19 pandemic in the United Kingdom (April 2020)."		
**4b-i) Report if outcomes were (self-)assessed through online questionnaires**		
"All participants were invited to fill out questionnaires via an online platform (Google Form) to assess their mental well-being on a weekly basis starting with the first day of the study (baseline)."<br><br>"All participants were required to self-report a range of validated measures of mental well-being (10-item Connor-Davidson Resilience scale [CD-RISC-10], 7-item Generalized Anxiety Disorder scale [GAD-7], Office of National Statistics Four Subjective Well-being Questions [ONS-4], World Health Organization-5 Well-Being Index [WHO-5]) and sleep (Minimal Insomnia Scale [MISS]) at baseline and at weeks 2 and 4. The self-reported measures of perceived stress (10-item Perceived Stress Score [PSS-10]) were obtained weekly."		
**4b-ii) Report how institutional affiliations are displayed**		
**5) CONSORT: Describe the interventions for each group with sufficient details to allow replication, including how and when they were actually administered**		
**5-i) Mention names, credential, affiliations of the developers, sponsors, and owners**		
**5-ii) Describe the history/development process**		
**5-iii) Revisions and updating**		
**5-iv) Quality assurance methods**		

5-v) Ensure replicability by publishing the source code, and/or providing screenshots/screen-capture video, and/or providing flowcharts of the algorithms used

5-vi) Digital preservation

https://apps.apple.com/ca/app/foundations-wellbeing-sleep/id1485424497

5-vii) Access

"After randomization, the intervention group received access and instructions to download the Foundations app."

Koa Health welcome-listed email addresses for the intervention arm and sent participants App access/log-in details.

5-viii) Mode of delivery, features/functionalities/components of the intervention and comparator, and the theoretical framework

"Foundations includes a plethora of interventions and psychoeducational content that are scientifically robust (ie, cognitive behavioral therapy [CBT], meditation)."

"The intervention app (Foundations) comprised interventions and psychoeducational content aimed at decreasing stress and promoting mental well-being (Figure 2). Content was organized into activities (ie, units of content) and could take a variety of formats, which are listed in Table 1. They all have a brief (typically 1-2 sentences) introduction and end with a closing sentence. Activities were either in the moment or part of a program. A program is a locked sequence of activities that is delivered in daily steps designed to teach a skill such as healthy sleep behaviors, positive psychology, working with thoughts, or relaxation techniques (see Table 2). In the moment activities were not part of a locked sequence of activities and could be accessed at any time. These activities included sleep meditations, articles, and mindfulness. Programs and activities were organized into a library of themed modules."

5-ix) Describe use parameters

"During the first 2 weeks of the study, the participants were required to perform a minimum number of activities and programs. In week 1, participants were instructed to complete the “Working with thoughts” program, consisting of journaling and CBT interventions focused on ameliorating cognitive distortions and unhelpful ruminative patterns, and a minimum of 4 free-choice activities. During week 2, participants had to complete 1 program of their choosing and a minimum of 4 other activities (free choice). During weeks 3 and 4, participants were free to use the app as little or as much as they wished with complete free choice of activities and programs."

5-x) Clarify the level of human involvement

5-xi) Report any prompts/reminders used

"For the first 2 weeks of the research period, participants were encouraged to use Foundations via daily text messages. The messages were written and sent by the messaging service WhatsApp and included wording such as:

Good morning! If you were able to start the programme yesterday then please do another activity of your choice from the Library page. If you weren’t able to start the programme yesterday, please do the first day of the Working with thoughts programme.
Remember, if you need to contact us at any point, the best way to get in touch is via email at: hello@evermind.health."

5-xii) Describe any co-interventions (incl. training/support)

Not applicable

6a) CONSORT: Completely defined pre-specified primary and secondary outcome measures, including how and when they were assessed

"All participants were invited to fill out questionnaires via an online platform (Google Form) to assess their mental well-being on a weekly basis starting with the first day of the study (baseline). All participants, regardless of group, were sent the link to the online platform via the messaging service WhatsApp at the same time. Perceived stress was assessed weekly, whereas all other measures were assessed every 2 weeks (ie, baseline, 2 weeks, and 4 weeks). All outcome measures were treated as continuous variables. Each measure and the associated questionnaire are detailed below.

Anxiety
Anxiety levels were assessed through the GAD-7 scale [20], a 7-item questionnaire that measures the severity of the subject’s anxiety over the previous week.
Sleep Problems
The MISS was used to examine sleep problems in the sample and their evolution across the study [21]. The MISS includes 3 items that cover issues of initiating sleep, waking up in the night, and not feeling refreshed in the morning.

Resilience
Resilience levels were assessed by the Connor-Davidson Resilience Scale (CD-RISC-10), which includes 10 items that assess the individual’s ability to cope successfully with adversity [22].

Mental Well-being
To assess current mental well-being, the World Health Organization-5 Well-Being Index (WHO-5) questionnaire was administered to the study participants. Each of the 5 items is scored with a Likert scale ranging from 0 to 5 (at no time, some of the time, less than half of the time, more than half of the time, most of the time, all of the time, respectively) [23].
In addition, the United Kingdom Office of National Statistics questions on well-being (ONS-4) scale was used to measure subjective well-being [24]. Each of the questions in this scale is aimed at measuring a different aspect of well-being: life satisfaction, worthwhileness, happiness, and anxiety, which are each rated by the subject from 0 (not at all) to 10 (completely). These questions are not designed to provide an aggregate score, but rather to illustrate different aspects of perceived well-being.

Perceived Stress
The degree to which participants perceived their life situations as stressful was assessed using the PSS-10 [18,19]. The time frame selected for questions was the past week, which enabled examination of weekly effects of the intervention. It should be
noted that participants were able to access the PSS-10 within the app whenever they liked. These additional data were not evaluated as part of the study."

6a-i) Online questionnaires: describe if they were validated for online use and apply CHERRIES items to describe how the questionnaires were designed/deployed

6a-ii) Describe whether and how “use” (including intensity of use/dosage) was defined/measured/monitored

1. App usage (average number of days)
2. " " (median days active)
3. " " (median minutes per day)
4. " " (average number of distinct activities and programs completed)

"The participants in the intervention group presented an average usage of the app of 18 days out of the 28 (SD 5.1) total days of the study period (median days active: 17). On average, participants engaged with the app 15.25 minutes per day (median minutes per day 13.93, SD 9.66). During the study period, users tried an average of 29 distinct activities and 3.9 programs."

6a-iii) Describe whether, how, and when qualitative feedback from participants was obtained

Not applicable

6b) CONSORT: Any changes to trial outcomes after the trial commenced, with reasons

"The study started at the peak of the COVID-19 pandemic in the United Kingdom (April 2020)."

7a) CONSORT: How sample size was determined

7a-i) Describe whether and how expected attrition was taken into account when calculating the sample size

"A power analysis based on published data and previous pilot studies [25] was performed (using the “pwr” R package) to estimate the required sample size for the study. The estimated sample size was at least 78 participants in each arm providing 0.8 power to detect an effect size of Cohen d=0.4 with an α of .
05"

7b) CONSORT: When applicable, explanation of any interim analyses and stopping guidelines

"All participants were invited to fill out questionnaires via an online platform (Google Form) to assess their mental well-being on a weekly basis starting with the first day of the study (baseline). All participants, regardless of group, were sent the link to the online platform via the messaging service WhatsApp at the same time. Perceived stress was assessed weekly, whereas all other measures were assessed every 2 weeks (ie, baseline, 2 weeks, and 4 weeks). All outcome measures were treated as continuous variables. Each measure and the associated questionnaire are detailed below.<br><br>Anxiety<br>Anxiety levels were assessed through the GAD-7 scale [20], a 7-item questionnaire that measures the severity of the subject's anxiety over the previous week.<br>Sleep Problems<br>The MISS was used to examine sleep problems in the sample and their evolution across the study [21]. The MISS includes 3 items that cover issues of initiating sleep, waking up in the night, and not feeling refreshed in the morning.<br><br>Resilience<br>Resilience levels were assessed by the Connor-Davidson Resilience Scale (CD-RISC-10), which includes 10 items that assess the individual's ability to cope successfully with adversity [22].<br><br>Mental Well-being<br>To assess current mental well-being, the World Health Organization-5 Well-Being Index (WHO-5) questionnaire was administered to the study participants. Each of the 5 items is scored with a Likert scale ranging from 0 to 5 (at no time, some of the time, less than half of the time, more than half of the time, most of the time, all of the time, respectively) [23].<br>In addition, the United Kingdom Office of National Statistics questions on well-being (ONS-4) scale was used to measure subjective well-being [24]. Each of the questions in this scale is aimed at measuring a different aspect of well-being: life satisfaction, worthwhileness, happiness, and anxiety, which are each rated by the subject from 0 (not at all) to 10 (completely). These questions are not designed to provide an aggregate score, but rather to illustrate different aspects of perceived well-being.<br><br>Perceived Stress<br>The degree to which participants perceived their life situations as stressful was assessed using the PSS-10 [18,19]. The time frame selected for questions was the past week, which enabled examination of weekly effects of the intervention. It should be<br>noted that participants were able to access the PSS-10 within the app whenever they liked. These additional data were not evaluated as part of the study."		
**8a) CONSORT: Method used to generate the random allocation sequence**		
"Eligible participants were randomly allocated (1:1) to either the intervention group or control group. Assignment of participants to the groups was performed with a computer-based algorithm (Python Software Foundation) that generated randomly permuted blocks, which were stratified by gender (male or female) and age (30-40 years or 40-50 years), and were balanced regarding the degree of perceived stress ("moderate perceived stress," PSS-10 score of 13-26, or "high perceived stress," PSS-10 score of 27-40) and sleep disturbances ("no sleep disturbances," MISS score of 0-4, or "moderate sleep disturbances," MISS score of 5-8). Furthermore, participants' baseline scores on the GAD-7 were analyzed for statistical differences and were rerandomized if needed."		
**8b) CONSORT: Type of randomisation; details of any restriction (such as blocking and block size)**		
"Eligible participants were randomly allocated (1:1) to either the intervention group or control group. Assignment of participants to the groups was performed with a computer-based algorithm (Python Software Foundation) that generated randomly permuted blocks."		
**9) CONSORT: Mechanism used to implement the random allocation sequence (such as sequentially numbered containers), describing any steps taken to conceal the sequence until interventions were assigned**		
"Eligible participants were randomly allocated (1:1) to either the intervention group or control group. Assignment of participants to the groups was performed with a computer-based algorithm (Python Software Foundation) that generated randomly permuted blocks, which were stratified by gender (male or female) and age (30-40 years or 40-50 years), and were balanced regarding the degree of perceived stress ("moderate perceived stress," PSS-10 score of 13-26, or "high perceived stress," PSS-10 score of 27-40) and sleep disturbances ("no sleep disturbances," MISS score of 0-4, or "moderate sleep disturbances," MISS score of 5-8). Furthermore, participants' baseline scores on the GAD-7 were analyzed for statistical differences and were rerandomized if needed."		
**10) CONSORT: Who generated the random allocation sequence, who enrolled participants, and who assigned participants to interventions**		
"Study participants were recruited through a research company specializing in study recruitment between the first 2 weeks of April"<br><br>"Eligible participants were randomly allocated (1:1) to either the intervention group or control group. Assignment of participants to the groups was performed with a computer-based algorithm (Python Software Foundation) that generated randomly permuted blocks, which were stratified by gender (male or female) and age (30-40 years or 40-50 years), and were balanced regarding the degree of perceived stress ("moderate perceived stress," PSS-10 score of 13-26, or "high perceived stress," PSS-10 score of 27-40) and sleep disturbances ("no sleep disturbances," MISS score of 0-4, or "moderate sleep disturbances," MISS score of 5-8). Furthermore, participants' baseline scores on the GAD-7 were analyzed for statistical differences and were rerandomized if needed. The randomization analysis was performed by a statistician who remained blinded to the identity of the groups."		
**11a) CONSORT: Blinding - If done, who was blinded after assignment to interventions (for example, participants, care providers, those assessing outcomes) and how**		
**11a-i) Specify who was blinded, and who wasn't**		
"Only the data analysts were blind to the group assignment. The control group participants were aware they were taking part in an intervention study, but were only given access to the app after the study (and were not informed they would have access until study completion)."		
**11a-ii) Discuss e.g., whether participants knew which intervention was the "intervention of interest" and which one was the "comparator"**		
Same answer as above		
**11b) CONSORT: If relevant, description of the similarity of interventions**		
Not applicable		
**12a) CONSORT: Statistical methods used to compare groups for primary and secondary outcomes**		
"Two principal sets of analyses were performed on each of the outcome measures. The first set of analyses, which we denote by within-group analyses, sought to determine whether there was a significant change within each group compared to the measure at the start of the study (baseline). Within-group paired two-tailed ttests were used for pre-post intervention assessments for each group. Statistical significance was set at P<.05. Bonferroni correction for multiple comparisons was performed adjusting the significance level to 1.25% for PSS-10 (significance level of 5% divided by 4 measures in time, baseline to weeks 1-4) and 2.5% (significance level of 5% divided by 2 measures in time, baseline to week 4) for the rest of the outcome measures.<br><br>The second set of analyses, denoted as between-group analyses, sought to determine whether the change from baseline (Δ) was equivalent in both groups. The analysis was performed using linear mixed models (LMMs) incorporating group (intervention or control), time, and group×time interaction terms, including a change score of 0 at the baseline time point and modeling participant as a random effect. Significance of the group variable was assessed using the likelihood ratio test. A confirmatory set of analyses was performed using an independent two-tailed t test on the differences of each group's scores in each measure at a given time point from their baseline scores (Δ). Statistical significance was set at P<.05."		
**12a-i) Imputation techniques to deal with attrition / missing values**		
"7 failed to complete the study (ie, did not use the app as required) and were excluded from the primary analysis. A further 9 participants (5 in the intervention group and 4 in the control group) were excluded due to missing data (failed to complete the outcome measure questionnaires)."		
**12b) CONSORT: Methods for additional analyses, such as subgroup analyses and adjusted analyses**		
"Additional analyses were performed with the factors of gender and age included on each of the outcome measures for both within-group and between-group comparisons."		
RESULTS		
**13a) CONSORT: For each group, the numbers of participants who were randomly assigned, received intended treatment, and were analysed for the primary outcome**		
Reported in Figure 3 (CONSORT flowchart of participants)		
**13b) CONSORT: For each group, losses and exclusions after randomisation, together with reasons**		
Reported in Figure 3 (CONSORT flowchart of participants). "From April 2020 to May 2020, 190 participants were enrolled in the study and randomized to either the intervention group (n=95) or control group (n=95). Of the 95 participants in the intervention group, 7 failed to complete the study (ie, did not use the app as required) and were excluded from the primary analysis. A further 9 participants (5 in the intervention group and 4 in the control group) were excluded due to missing data (failed to complete the outcome measure questionnaires). An additional 38 participants (21 in the intervention group and 17 in the control group) were excluded from the analysis due to a calculation error of the PSS-10 at screening (PSS-10 score<13). Due to the study's single-blind design, this error was not identified until after the study was completed. Of the remaining participants, 74 (79%) from the control group and 62 (65%) from the intervention group completed the study and were<br>analyzed for the primary outcome and secondary outcome measures."		
**13b-i) Attrition diagram**		
**14a) CONSORT: Dates defining the periods of recruitment and follow-up**		
"The study started on April 22, 2020, and ended on May 20, 2020."		
**14a-i) Indicate if critical "secular events" fell into the study period**		
**14b) CONSORT: Why the trial ended or was stopped (early)**		
The trial ended within the intended time period		
**15) CONSORT: A table showing baseline demographic and clinical characteristics for each group**		

Reported in Table 3 (characteristics of study participants at baseline)		
**15-i) Report demographics associated with digital divide issues**		
Not included in the present study (excluding age and gender of the participants)		
**16a) CONSORT: For each group, number of participants (denominator) included in each analysis and whether the analysis was by original assigned groups**		
**16-i) Report multiple "denominators" and provide definitions**		
Reported in Figure 3 (CONSORT flowchart of participants)		
**16-ii) Primary analysis should be intent-to-treat**		
**17a) CONSORT: For each primary and secondary outcome, results for each group, and the estimated effect size and its precision (such as 95% confidence interval)**		
Information reported in Tables 4 and 5		
**17a-i) Presentation of process outcomes such as metrics of use and intensity of use**		
"The participants in the intervention group presented an average usage of the app of 18 days out of the 28 (SD 5.1) total days of the study period (median days active: 17). On average, participants engaged with the app 15.25 minutes per day (median minutes per day 13.93, SD 9.66). During the study period, users tried an average of 29 distinct activities and 3.9 programs. However, no correlation was found between the total amount of engagement with the app and the difference from baseline to 4 weeks in any of the outcome measures (all P>.05)."		
**17b) CONSORT: For binary outcomes, presentation of both absolute and relative effect sizes is recommended**		
Not applicable		
**18) CONSORT: Results of any other analyses performed, including subgroup analyses and adjusted analyses, distinguishing pre-specified from exploratory**		
"Additional analyses were performed with the factors of gender and age included on each of the outcome measures for both within-group and between-group comparisons. No statistically significant differences were observed, due to the fact that the size of groups was small."		
**18-i) Subgroup analysis of comparing only users**		
**19) CONSORT: All important harms or unintended effects in each group**		
No serious adverse effects or significant deterioration in mental wellbeing were observed for any measure.		
**19-i) Include privacy breaches, technical problems**		
"An additional 38 participants (21 in the intervention group and 17 in the control group) were excluded from the analysis due to a calculation error of the PSS-10 at screening (PSS-10 score<13)."		
**19-ii) Include qualitative feedback from participants or observations from staff/researchers**		
DISCUSSION		
**20) CONSORT: Trial limitations, addressing sources of potential bias, imprecision, multiplicity of analyses**		
**20-i) Typical limitations in ehealth trials**		
"Several important limitations of this study should be recognized. Perhaps the most important limitation is the notable number of participants excluded from the primary analysis due to a calculation error of the PSS-10 at screening. It remains uncertain whether a larger sample of patients would result in outcomes that differ from those observed; however, the sample size for the final analysis (N=136) is comparable to those reported in the literature on digital interventions for mental well-being [27,28].<br><br>Second, it is possible that the daily scheduled broadcast messages received by participants in the Foundations group contributed to a placebo effect or to results that do not reflect real-world usage of the app. Conversely, it is also possible that the frequency of the notifications hindered engagement in some cases, as the amount of contact for optimizing user engagement likely varies across participants. However, it is notable that the size of the effect was greater at 4 weeks than at 2 weeks for all measures. Participants received no messages during weeks 3 and 4 other than the links to fill out the questionnaires. It therefore seems unlikely that the daily messaging could account for the efficacy observed in the study. However, the impact of the frequency and content of notifications on well-being outcomes remains to be fully elucidated and further studies may be warranted.<br><br>Another limitation of the study is the use of a monetary incentive for participation. Previous studies have shown that monetary incentives can increase engagement with wellness apps yet have no impact on the outcome of the study [12]. However, the true influence of the monetary incentive in this case is unknown.<br><br>Finally, it is necessary to acknowledge the limitations of a single-blind design, as used here. Only the data analysts were blind to the group assignment. The control group participants were aware they were taking part in an intervention study, but were only given access to the app after the study (and were not informed they would have access until study completion). The design of the study and the passive nature of the control group do not allow ruling out digital placebo effects in the intervention group derived from their expectations about the interventions [36]. Both groups, however, received the same messages and invitations to fill out the outcome questionnaires. It remains possible that insights into mental well-being through completion of the questionnaires, along with knowledge that they were participating in an intervention trial may have impacted outcome measures. Future studies may explore the use of alternative."		
**21) CONSORT: Generalisability (external validity, applicability) of the trial findings**		
**21-i) Generalizability to other populations**		
"Although this study examined the efficacy of Foundations in a working population, a truly rigorous investigation of its effects on workplace mental health would require a future study employing randomized, controlled allocation of participants to an intervention arm (Foundations) or placebo arm, both in the absence and presence of employment. At the very least, these preliminary observations provide evidence that working adults using the Foundations app can experience significant improvement in their mental well-being during a 2-week period."		
**21-ii) Discuss if there were elements in the RCT that would be different in a routine application setting**		
"For the first 2 weeks of the research period, participants were encouraged to use Foundations via daily text messages." This differs from real world practice, as users would not receive reminders via text. A minimum level of engagement with the app would also not be enforced/recommended outside of a research setting.		
**22) CONSORT: Interpretation consistent with results, balancing benefits and harms, and considering other relevant evidence**		
**22-i) Restate study questions and summarize the answers suggested by the data, starting with primary outcomes and process outcomes (use)**		
"The aim of the study was to investigate the efficacy of a mobile app, Foundations, in improving mental well-being in adults with moderate to high levels of stress and anxiety. Given the timing of the study (April 2020 to May 2020), our secondary aim was to assess the efficacy of Foundations in mitigating the mental health challenges surrounding the COVID-19 pandemic.<br><br>Encouragingly, results of this proof-of-principle study confirmed four out of five of our hypotheses by demonstrating that the use of Foundations can significantly improve measures of (1) anxiety, (2) resilience, (3) well-being, and (4) sleep relative to a control group within 2 weeks of use, with greater effects after 4 weeks. In contrast to our final hypothesis, perceived stress was reduced within the intervention group, although the results did not reach statistical significance relative to the control group."		
**22-ii) Highlight unanswered new questions, suggest future research**		

"Before we can optimize the efficacy of digital health technologies such as Foundations, we must build a richer understanding of which interventions are most effective for which individual needs. It was not feasible with the sample size of the current study to evaluate the efficacy of individual components of the app with respect to symptom type and severity, and the content that the participant engaged with during the course of the study. An important question for future research is whether personalization of these interventions makes care more effective.<br><br>Similarly, it is unclear whether there was an effect of the level of engagement (ie, a dose effect). Although a satisfactory level of engagement was observed in this study, establishing a dose-response relationship between usage and improvement in mental well-being could prove useful for intervention personalization and bears further investigation in future.<br>A specific domain that has gained attention in recent years is that of work-related mental health. This is due to an increase of awareness of the magnitude and costs of this issue.<br><br>Concretely, it has been reported that an outstanding 72% of employees of large organizations in the United Kingdom have disclosed an increase of CMD during 2019 [37]. Ill mental health in the workplace is associated with decreased productivity, early retirement, increased sickness absence, presenteeism (not working at capacity while at work), and staff turnover. All this translates into an estimated cost of over US $45 billion per year for companies, which has increased by 16% in the last few years [37]. Although this study examined the efficacy of Foundations in a working population, a truly rigorous investigation of its effects on workplace mental health would require a future study employing randomized, controlled allocation of participants to an intervention arm (Foundations) or placebo arm, both in the absence and presence of employment. At the very least, these preliminary observations provide evidence that working adults using the Foundations app can experience significant improvement in their mental well-being during a 2-week period.<br><br>Future research is also required to evaluate the long-term effects of Foundations on mental well-being both in terms of postintervention follow-up and longer sustained use of the app. Looking forward and if replicated in further studies, these results may have important implications for addressing the treatment gap in mental health care through the use of evidence-based digital interventions.<br><br>Overall, results from this study may help to propel the use of mobile apps such as Foundations to assume a more widespread role in both the promotion and maintenance of mental health. This could offer new possibilities to further optimize the efficacy of these technologies while removing obstacles for evidence-based mental health care."		
Other information		
**23) CONSORT: Registration number and name of trial registry**		
"Due to unforeseen logistical reasons, the study was not preregistered; however, all statistical testing was performed by a data analyst who remained blind to treatment assignment. Furthermore, the authors confirm that all ongoing studies for the Foundations app are registered."		
**24) CONSORT: Where the full trial protocol can be accessed, if available**		
The full trial protocol is not openly accessible to the general public		
**25) CONSORT: Sources of funding and other support (such as supply of drugs), role of funders**		
The study was self-funded (Koa Health)		
**X26-i) Comment on ethics committee approval**		
**x26-ii) Outline informed consent procedures**		
**X26-iii) Safety and security procedures**		
**X27-i) State the relation of the study team towards the system being evaluated**		
"All authors, excluding NRH and LS, were employees of Telefónica Innovación Alpha at the time of the study. The app and the authors have now transferred from Telefonica Innovation Alpha to Koa Health."		
